# Supplementary material for: Discovery of Bactericidal Proteins from Staphylococcus Phage Stab21 Using a High-Throughput Screening Method
Source: Antibiotics (Basel). 2023 Jul 21;12(7):1213. doi: 10.3390/antibiotics12071213 (PMC10376165; doi:10.3390/antibiotics12071213)
Supplement: Supplementary file 1 [file antibiotics-12-01213-s001.zip › Figure S2.pdf]

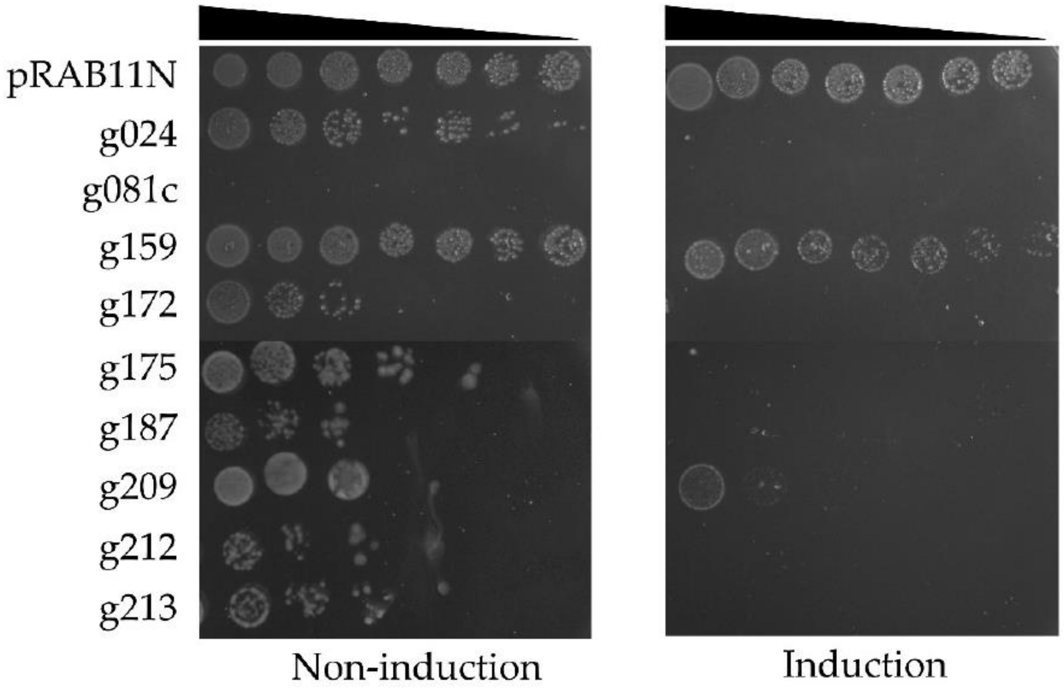


**Figure S2.** Toxicity drop test of *E. coli* DH10B/pRAB11N-HPUFs. Genes under non-inducing conditions in the absence of anhydrotetracycline (ATc) and genes inducing conditions in the presence of ATc (0.4 μM).
