# Supplementary material for: Discovery of Bactericidal Proteins from Staphylococcus Phage Stab21 Using a High-Throughput Screening Method
Source: Antibiotics (Basel). 2023 Jul 21;12(7):1213. doi: 10.3390/antibiotics12071213 (PMC10376165; doi:10.3390/antibiotics12071213)
Supplement: Supplementary file 1 [file antibiotics-12-01213-s001.zip › Figure S3.pdf]

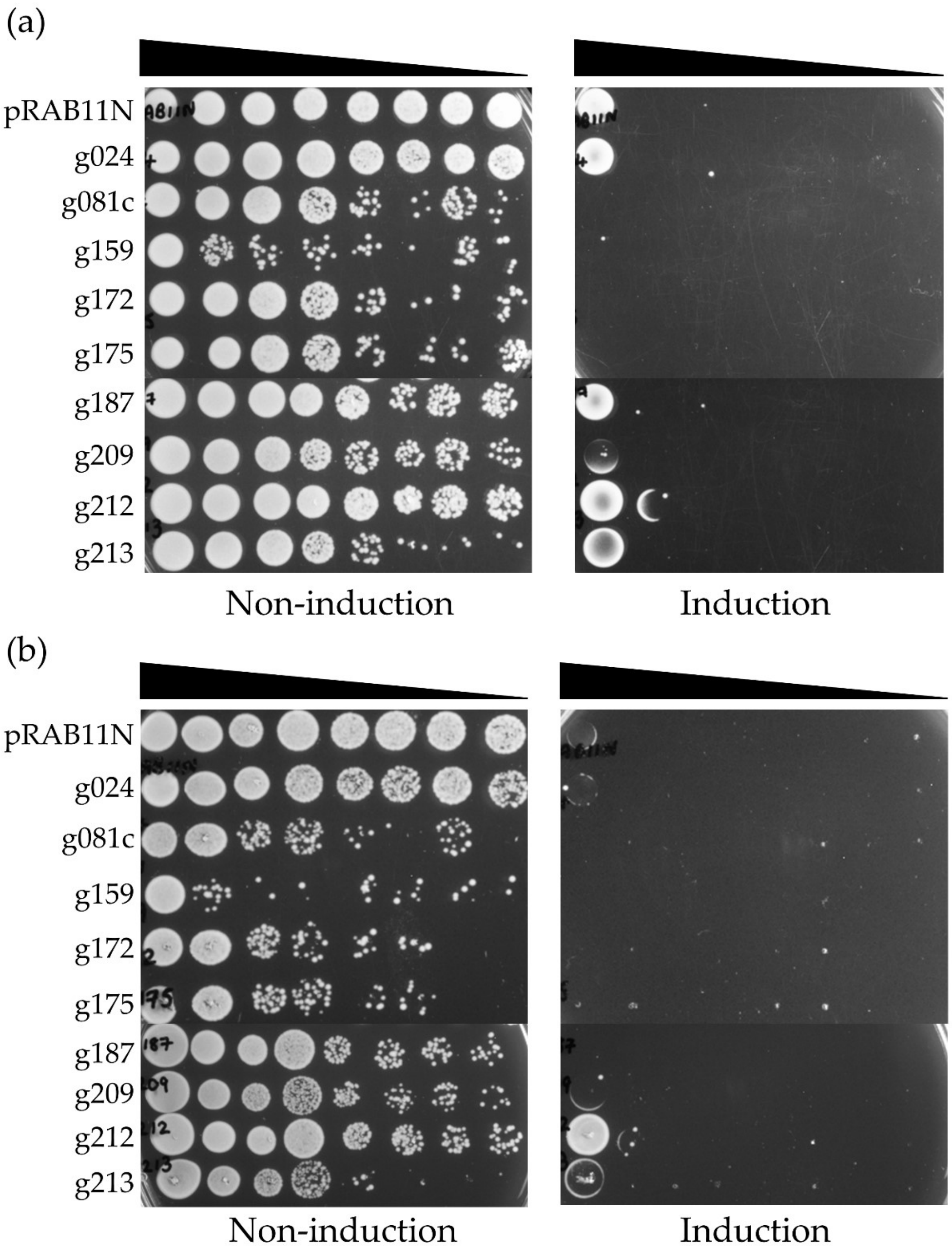

Figure S3. Toxicity drop test of *S. aureus* RN4220/pRAB11N-HPUFs on various concentrations of antibiotic. (a) Genes under non-inducing conditions using chloramphenicol 30 μg/mL (Cm30) and genes under inducing conditions using Cm30 and anhydrotetracycline (ATc) 0.4μM. (b) Genes under non-inducing conditions using chloramphenicol 10 μg/mL (Cm10) and genes under inducing conditions using Cm10 and ATc 0.4μM.
