# Supplementary material for: Discovery of Bactericidal Proteins from Staphylococcus Phage Stab21 Using a High-Throughput Screening Method
Source: Antibiotics (Basel). 2023 Jul 21;12(7):1213. doi: 10.3390/antibiotics12071213 (PMC10376165; doi:10.3390/antibiotics12071213)
Supplement: Supplementary file 1 [file antibiotics-12-01213-s001.zip › Figure S4.pdf]

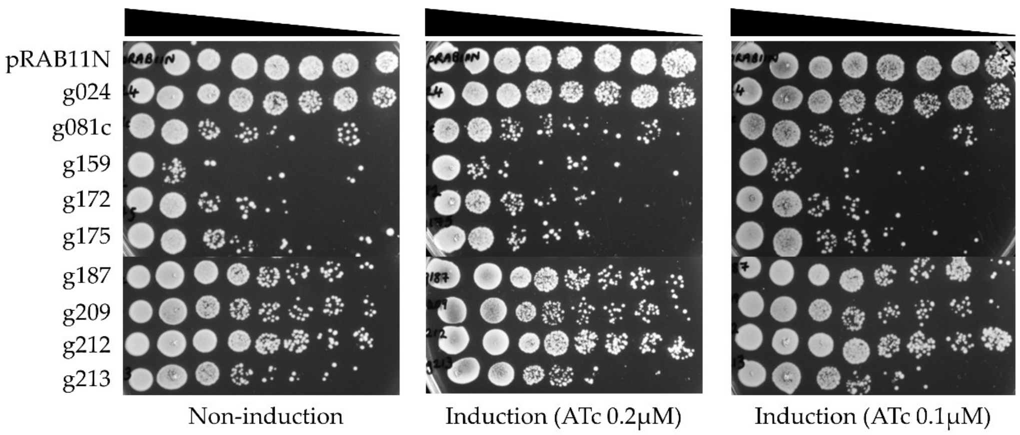

Figure S4. Toxicity drop test of *S. aureus* RN4220/pRAB11N-HPUFs on different concentrations of inducer. Genes under non-inducing conditions using chloramphenicol 30 μg/mL (Cm30) and genes under inducing conditions using Cm30 and either anhydrotetracycline (ATc) 0.2μM or ATc 0.1μM.
