# Supplementary material for: Discovery of Bactericidal Proteins from Staphylococcus Phage Stab21 Using a High-Throughput Screening Method
Source: Antibiotics (Basel). 2023 Jul 21;12(7):1213. doi: 10.3390/antibiotics12071213 (PMC10376165; doi:10.3390/antibiotics12071213)
Supplement: Supplementary file 1 [file antibiotics-12-01213-s001.zip › Figure S5.pdf]

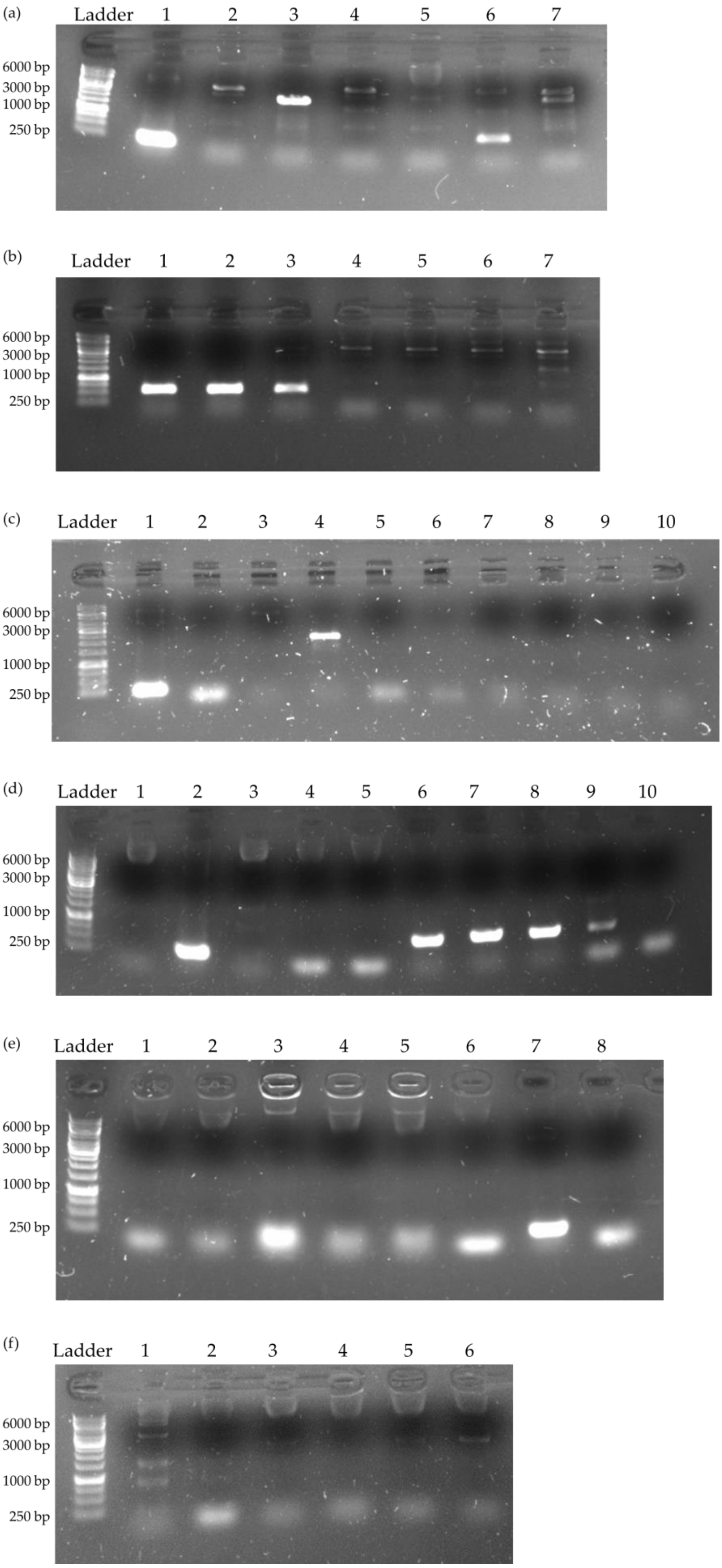
(a)
Ladder 1 2 3 4 5 6 7
6000 bp
3000 bp
1000 bp
250 bp
(b)
Ladder 1 2 3 4 5 6 7
6000 bp
3000 bp
1000 bp
250 bp
(c)
Ladder 1 2 3 4 5 6 7 8 9 10
6000 bp
3000 bp
1000 bp
250 bp
(d)
Ladder 1 2 3 4 5 6 7 8 9 10
6000 bp
3000 bp
1000 bp
250 bp
(e)
Ladder 1 2 3 4 5 6 7 8
6000 bp
3000 bp
1000 bp
250 bp
(f)
Ladder 1 2 3 4 5 6
6000 bp
3000 bp
1000 bp
250 bp

Figure S5. Gel electrophoresis of *E. coli* DH10B/pRAB11N-toxHPUFs ATc tolerant mutant colony PCR products. (a) Lane 1 is a positive PCR control with empty pRAB11N, lane 2-4 are from pRAB11N-g024-T tolerant colonies, and lane 5-7 are from pRAB11N-g172-T colonies; (b) Lanes 1-3 are from pRAB11N-g187-T tolerant mutants and lanes 4-7 are from pRAB11N-g213-T; (c) Lane 1 is pRAB11N, lane 2 is a negative PCR control with water, lanes 3-6 are from pRAB11N-g024-T, and lanes 7-10 are from pRAB11N-g213-T; (d) Lane 1 is a negative PCR control with water, lane 2 is a positive control with pRAB11N, lanes 3-5 are from pRAB11N-g024-T, and lanes 6-9 are from pRAB11N-g187-T, and lane 10 is from pRAB11N-g213-T; (e) Lanes 1-6 are representing pRAB11N-g081c-T, lane 7 is pRAB11N and lane 8 is negative PCR control with water; (f) Lanes 1-2 are pRAB11N-*g213*-T, and lanes 3-6 are pRAB11N-*g081c*-T.
