# Supplementary material for: Discovery of Bactericidal Proteins from Staphylococcus Phage Stab21 Using a High-Throughput Screening Method
Source: Antibiotics (Basel). 2023 Jul 21;12(7):1213. doi: 10.3390/antibiotics12071213 (PMC10376165; doi:10.3390/antibiotics12071213)
Supplement: Supplementary file 1 [file antibiotics-12-01213-s001.zip › Figure S6.pdf]

(a)

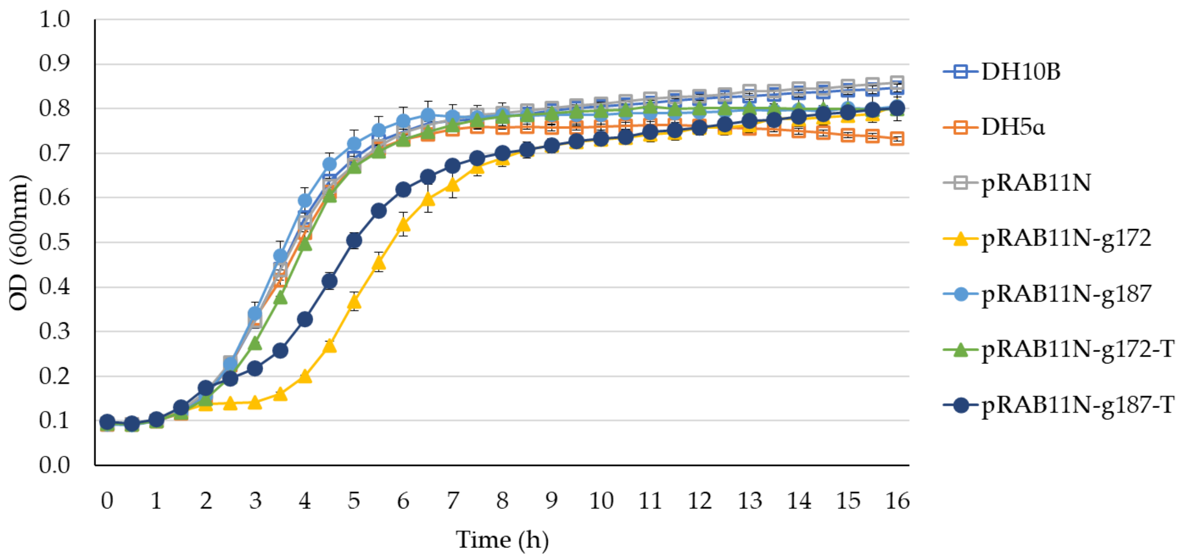

(b)

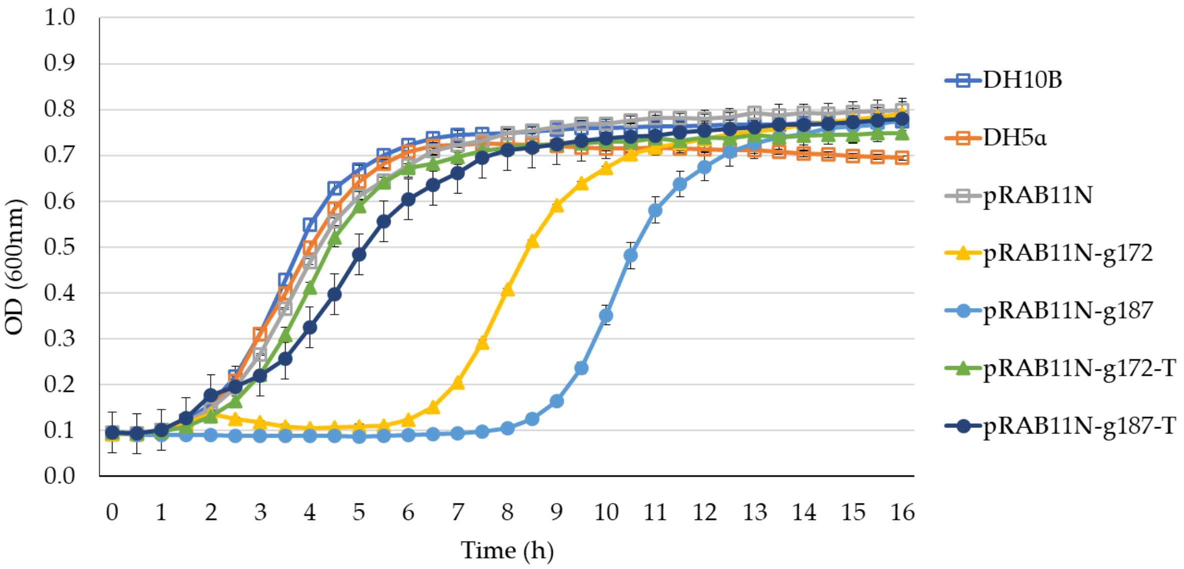

Figure S6. ATc sensitive and tolerant *E. coli* DH10B/pRAB11N-toxHPUF toxicity screening. (a) Toxicity screening without anhydrotetracycline (ATc) induction; (b) Toxicity screening with ATc induction. Error bars show ± standard deviation calculated using triplicate results. OD, optical density.
