# Supplementary material for: Discovery of Bactericidal Proteins from Staphylococcus Phage Stab21 Using a High-Throughput Screening Method
Source: Antibiotics (Basel). 2023 Jul 21;12(7):1213. doi: 10.3390/antibiotics12071213 (PMC10376165; doi:10.3390/antibiotics12071213)
Supplement: Supplementary file 1 [file antibiotics-12-01213-s001.zip › Figure S7.pdf]

```
g172        MSRLDKSKVINSALELLNEVGIEGLTTRKLAQKLGVEQPTLYWHVKNKRALLDALAIEML     60
g172T       MSRLDKSKVINSALELLNEVGIEGLTTRKLAQKLGVEQPTLYWHVKNKRALLDALAIEML     60
g187        MSRLDKSKVINSALELLNEVGIEGLTTRKLAQKLGVEQPTLYWHVKNKRALLDALAIEML     60
g187T       MSRLDKSKVINSALELLNEVGIEGLTTRKLAQKLGVEQPTLYWHVKNKRALLDALAIEML     60
            ************************************************************

g172        DRHHTHFCPLEGESWQDFLRNNAKSFRCALLSHRDGAKVHLGTRPTEKQYETLENQLAFL     120
g172T       DRHHTHFCPLEGESWQDFLRNNAKSFRCALLSHRDGAKVHLGTRPTEKQYETRENQLAFL     120
g187        DRHHTHFCPLEGESWQDFLRNNAKSFRCALLSHRDGAKVHLGTRPTEKQYETLENQLAFL     120
g187T       DRHHTHFCPLEGESWQDFLRNKAKSFRCALLSHRDGAKVHLGTRPTEKQYETLENQLAFL     120
            *********************:****************************** *******

g172        CQQGFSLENALYALSAVGHFTLGCVLEDQEHQVAKEERETPTTDSMPPLLRQAIELFDHQ     180
g172T       CQQGFSLENALYALSAVGHFTLGCVLEDQEHQVAKEERETPTTDSMPPLLRQAIELFDHQ     180
g187        CQQGFSLENALYALSAVGHFTLGCVLEDQEHQVAKEERETPTTDSMPPLLRQAIELFDHQ     180
g187T       CQQGFSLENALYALSAVGHFTLGCVLEDQEHQVAKEERETPTTDSMPPLLRQAIELFDHQ     180
            ************************************************************

g172        GAEPAFLFGLELIICGLEKQLKCESGS 207
g172T       GAEPAFLFGLELIICGLEKQLKCESGS 207
g187        GAEPAFLFGLELIICGLEKQLKCESGS 207
g187T       GAEPAFLFGLELIICGLEKQLKCESGS 207
            ***************************
```

Figure S7. TetR sequence alignment of ATc sensitive pRAB11N-*g172*, *g187* and ATc tolerant pRAB11N-*g172*-T, *g187*-T. Amino acid of interest highlighted in yellow.
