# Supplementary material for: Discovery of Bactericidal Proteins from Staphylococcus Phage Stab21 Using a High-Throughput Screening Method
Source: Antibiotics (Basel). 2023 Jul 21;12(7):1213. doi: 10.3390/antibiotics12071213 (PMC10376165; doi:10.3390/antibiotics12071213)
Supplement: Supplementary file 1 [file antibiotics-12-01213-s001.zip › Figure S8.pdf]

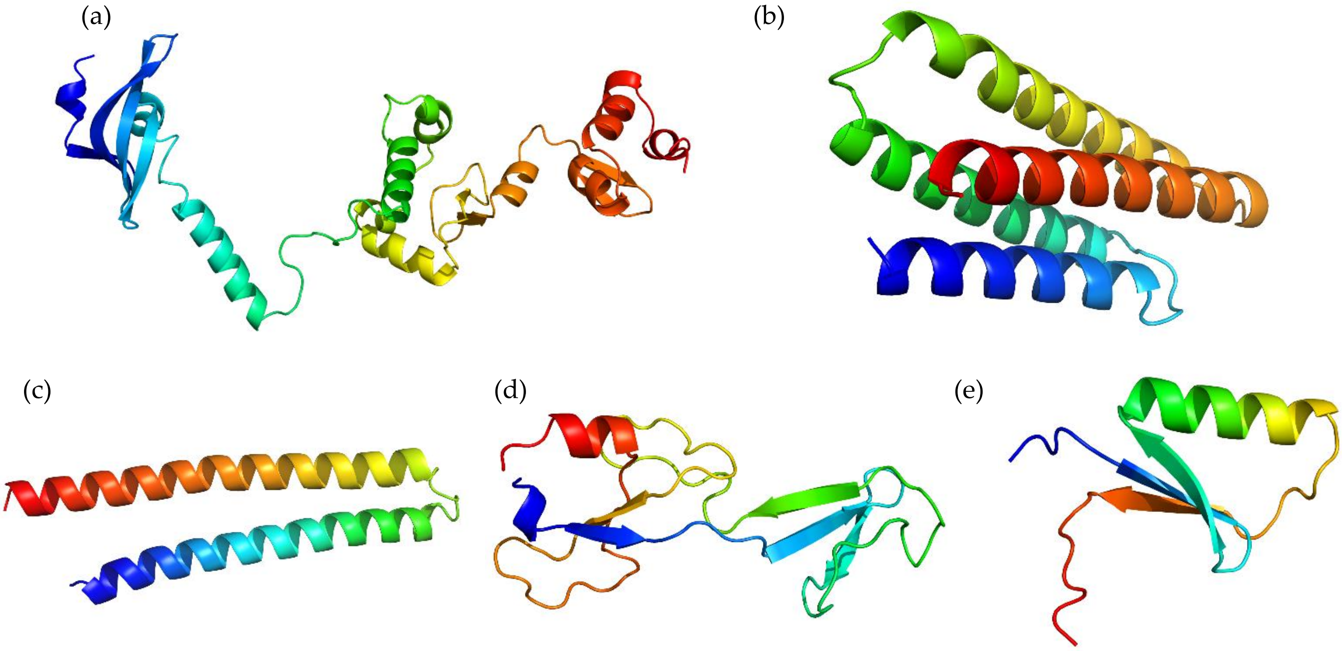

Figure S8. Predicted structures of the top 5 toxHPUFs (a) Gp024, (b) Gp081c, (c) Gp172, (d) Gp187 and (e) Gp213 generated by AlphaFold2. N-termini in blue and C-termini in red.
