# Supplementary material for: Discovery of Bactericidal Proteins from Staphylococcus Phage Stab21 Using a High-Throughput Screening Method
Source: Antibiotics (Basel). 2023 Jul 21;12(7):1213. doi: 10.3390/antibiotics12071213 (PMC10376165; doi:10.3390/antibiotics12071213)
Supplement: Supplementary file 1 [file antibiotics-12-01213-s001.zip › Figure S9.pdf]

(a) (b)

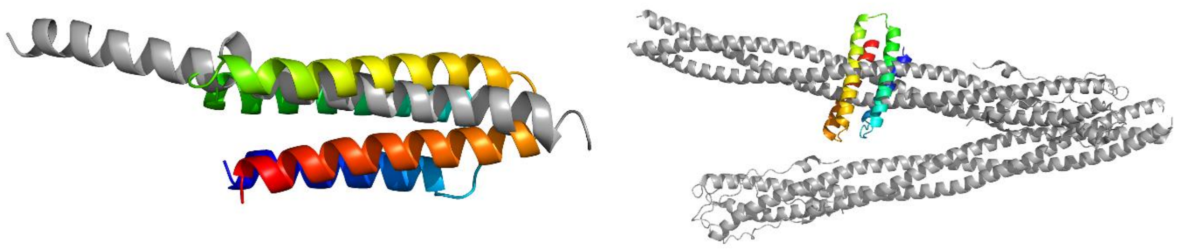

Figure S9. Superimposition of Gp081c (rainbow, N-terminus blue, C-terminus red) with (a) the top Phyre2 model of *Saccharomyces cerevisiae* monopolin complex protein subunit CSM1 (grey) and (b) the best hit of HHPred functional models of *Enterobacter* phage 22 tail needle protein Gp26 (grey).
