# Supplementary material for: Discovery of Bactericidal Proteins from Staphylococcus Phage Stab21 Using a High-Throughput Screening Method
Source: Antibiotics (Basel). 2023 Jul 21;12(7):1213. doi: 10.3390/antibiotics12071213 (PMC10376165; doi:10.3390/antibiotics12071213)
Supplement: Supplementary file 1 [file antibiotics-12-01213-s001.zip › Table S1.pdf]

Table S1. Details of the NGS assay. For each gene, two pairs of complementary sequences over the ligation joints were determined *in silico* containing 10-11 nucleotides of the gene (flanked with start and stop codon) and plasmid specific sequences. The joints are named with V, vector; G, gene fragment; F, forward; and R, reverse, indicating the directions. The joint-read totals for each gene and their percentages of total joint-reads were calculated. These were then used to calculate the plasmid DNA ligation mixture abundance ratios.

Pool	Gene	Joint	Joint Sequence	Pooled ligation mixture			Pooled plasmid DNA			Plasmid/ligation ratio
				N joint-reads	Gene total	Gene %	*N* joint-reads	Gene total	Gene %	
1	*g002*	VGF	ATGCCTGCAG**GCGGCCGC**ATGATAATAA	0			0			
		VGR	TTATTATCAT**GCGGCCGC**CTGCAGGCAT	0			23			
		GVF	AATTCTTATAG**GCTAGC**TCTAGAGGATC	0			0			
		GVR	GATCCTCTAGA**GCTAGC**CTATAAGAATT	0	0	0.0000	0	23	0.0036	-
	g003	VGF	ATGCCTGCAG**GCGGCCGC**ATGATTGATA	1730			18305			
		VGR	TATCAATCAT**GCGGCCGC**CTGCAGGCAT	16			14114			
		GVF	AGATATTTTAA**GCTAGC**TCTAGAGGATC	118			16608			
		GVR	GATCCTCTAGA**GCTAGC**TTAAAATATCT	2206	4070	9.7964	16031	65058	10.0636	1.0273
	g005	VGF	ATGCCTGCAG**GCGGCCGC**ATGATAGAAA	2011			13464			
		VGR	TTTCTATCAT**GCGGCCGC**CTGCAGGCAT	24			10057			
		GVF	TAGATTATTAG**GCTAGC**TCTAGAGGATC	88			11375			
		GVR	GATCCTCTAGA**GCTAGC**CTAATAATCTA	2170	4293	10.3331	12502	47398	7.3318	0.7095
	g006	VGF	ATGCCTGCAG**GCGGCCGC**ATGATAGAGA	1443			14081			
		VGR	TCTCTATCAT**GCGGCCGC**CTGCAGGCAT	9			11108			
		GVF	AGGAGATGTAA**GCTAGC**TCTAGAGGATC	153			13754			
		GVR	GATCCTCTAGA**GCTAGC**TTACATCTCCT	1969	3574	8.6025	14576	53519	8.2787	0.9624
	g008	VGF	ATGCCTGCAG**GCGGCCGC**ATGGTTACTT	342			1058			
		VGR	AAGTAACCAT**GCGGCCGC**CTGCAGGCAT	380			948			
		GVF	ACGTAGGATAG**GGTACC**GAGCTCGAATT	1619			1205			
		GVR	AATTCGAGCTC**GGTACC**CTATCCTACGT	2805	5146	12.3863	1014	4225	0.6535	0.0528
	g009	VGF	ATGCCTGCAG**GCGGCCGC**ATGATAGTTA	1984			19842			
		VGR	TAACTATCAT**GCGGCCGC**CTGCAGGCAT	12			15330			
		GVF	GCGGGGATTGA**GCTAGC**TCTAGAGGATC	117			16929			
		GVR	GATCCTCTAGA**GCTAGC**TCAATCCCCGC	2452	4565	10.9878	20046	72147	11.1601	1.0157
	g010c	VGF	ATGCCTGCAG**GCGGCCGC**ATGAAAATAA	1380			17371			
		VGR	TTATTTTCAT**GCGGCCGC**CTGCAGGCAT	5			13694			
		GVF	ATATTCTATAA**GCTAGC**TCTAGAGGATC	52	2864	6.8936	15546	62033	9.5957	1.3920

	GVR	GATCCTCTAGA**GCTAGC**TTATAGAATAT	1427			15422			
g012	VGF	ATGCCTGCAG**GCGGCCGC**ATGACAAACA	1994			18602			
	VGR	TGTTTGTCAT**GCGGCCGC**CTGCAGGCAT	11			10041			
	GVF	TTAAGAATTAA**GCTAGC**TCTAGAGGATC	125			16569			
	GVR	GATCCTCTAGA**GCTAGC**TTAATTCTTAA	2100	4230	10.1815	15189	60401	9.3432	0.9177
g013	VGF	ATGCCTGCAG**GCGGCCGC**ATGATATTAG	893			14591			
	VGR	CTAATATCAT**GCGGCCGC**CTGCAGGCAT	8			11736			
	GVF	AAACTAAATAA**GCTAGC**TCTAGAGGATC	91			12334			
	GVR	GATCCTCTAGA**GCTAGC**TTATTTAGTTT	1043	2035	4.8982	12546	51207	7.9210	1.6171
g017	VGF	ATGCCTGCAG**GCGGCCGC**ATGAAAATGT	1640			11934			
	VGR	ACATTTTCAT**GCGGCCGC**CTGCAGGCAT	11			8705			
	GVF	TCAGACATTGA**GCTAGC**TCTAGAGGATC	58			10723			
	GVR	GATCCTCTAGA**GCTAGC**TCAATGTCTGA	1797	3506	8.4388	11823	43185	6.6801	0.7916
g018	VGF	ATGCCTGCAG**GCGGCCGC**ATGAACAGAT	0			0			
	VGR	ATCTGTTCAT**GCGGCCGC**CTGCAGGCAT	0			0			
	GVF	AATACGCATAA**GCTAGC**TCTAGAGGATC	0			0			
	GVR	GATCCTCTAGA**GCTAGC**TTATGCGTATT	0	0	0.0000	0	0	0.0000	-
g020	VGF	ATGCCTGCAG**GCGGCCGC**ATGATTACAA	183			13515			
	VGR	TTGTAATCAT**GCGGCCGC**CTGCAGGCAT	15			11481			
	GVF	AAACTGTTTAA**GCTAGC**TCTAGAGGATC	48			14301			
	GVR	GATCCTCTAGA**GCTAGC**TTAAACAGTTT	307	553	1.3311	12171	51468	7.9614	5.9813
g021	VGF	ATGCCTGCAG**GCGGCCGC**ATGACAAATA	1264			7618			
	VGR	TATTTGTCAT**GCGGCCGC**CTGCAGGCAT	7			4485			
	GVF	TGGCACTGTAG**GCTAGC**TCTAGAGGATC	143			5766			
	GVR	GATCCTCTAGA**GCTAGC**CTACAGTGCCA	895	2309	5.5577	6800	24669	3.8160	0.6866
g022	VGF	ATGCCTGCAG**GCGGCCGC**ATGTTGAAGA	170			13327			
	VGR	TCTTCAACAT**GCGGCCGC**CTGCAGGCAT	9			12253			
	GVF	AAGAAATGTAA**GCTAGC**TCTAGAGGATC	120			14251			
	GVR	GATCCTCTAGA**GCTAGC**TTACATTTCTT	473	772	1.8582	13093	52924	8.1866	4.4057
g024	VGF	ATGCCTGCAG**GCGGCCGC**ATGAACATTA	1510			5473			
	VGR	TAATGTTCAT**GCGGCCGC**CTGCAGGCAT	9			4309			
	GVF	TAGGGAGGTAG**GCTAGC**TCTAGAGGATC	63			5354			
	GVR	GATCCTCTAGA**GCTAGC**CTACCTCCCTA	1491	3073	7.3966	5106	20242	3.1312	0.4233
g026	VGF	ATGCCTGCAG**GCGGCCGC**ATGATGAACA	144			9578			
	VGR	TGTTCATCAT**GCGGCCGC**CTGCAGGCAT	2	556	1.3383	8994	37971	5.8736	4.3889

		GVF	GAATATTTTAA**GCTAGC**TCTAGAGGATC	97			11175			
		GVR	GATCCTCTAGA**GCTAGC**TTAAAATATTC	313			8224			
	Total			41546			646470			
2	*g027c*	VGF	ATGCCTGCAG**GCGGCCGC**ATGAAACTAT	299			5930			
		VGR	ATAGTTTCAT**GCGGCCGC**CTGCAGGCAT	2			6819			
		GVF	ATAATACCTAA**GCTAGC**TCTAGAGGATC	357			7588			
		GVR	GATCCTCTAGA**GCTAGC**TTAGGTATTAT	845	1503	5.3108	5288	25625	5.4160	1.0198
	g028c	VGF	ATGCCTGCAG**GCGGCCGC**ATGAACAAAG	921			8265			
		VGR	CTTTGTTCAT**GCGGCCGC**CTGCAGGCAT	2			9143			
		GVF	AGAATAAGTAA**GCTAGC**TCTAGAGGATC	122			10759			
		GVR	GATCCTCTAGA**GCTAGC**TTACTTATTCT	1032	2077	7.3390	8548	36715	7.7599	1.0574
	g029c	VGF	ATGCCTGCAG**GCGGCCGC**ATGGGATTAG	747			4673			
		VGR	CTAATCCCAT**GCGGCCGC**CTGCAGGCAT	3			5114			
		GVF	TAAATGTATAA**GCTAGC**TCTAGAGGATC	89			5773			
		GVR	GATCCTCTAGA**GCTAGC**TTATACATTTA	981	1820	6.4309	4046	19606	4.1438	0.6444
	g030c	VGF	ATGCCTGCAG**GCGGCCGC**ATGGAAAATT	867			4584			
		VGR	AATTTTCCAT**GCGGCCGC**CTGCAGGCAT	7			5282			
		GVF	AGGAAAAATAA**GCTAGC**TCTAGAGGATC	85			6289			
		GVR	GATCCTCTAGA**GCTAGC**TTATTTTTCCT	975	1934	6.8337	4678	20833	4.4032	0.6443
	g031c	VGF	ATGCCTGCAG**GCGGCCGC**ATGAGATATG	568			3391			
		VGR	CATATCTCAT**GCGGCCGC**CTGCAGGCAT	3			3936			
		GVF	AATCACAATGA**GCTAGC**TCTAGAGGATC	103			4924			
		GVR	GATCCTCTAGA**GCTAGC**TCATTGTGATT	1170	1844	6.5157	3363	15614	3.3001	0.5065
	g033c	VGF	ATGCCTGCAG**GCGGCCGC**ATGAATATCA	878			11930			
		VGR	TGATATTCAT**GCGGCCGC**CTGCAGGCAT	3			11898			
		GVF	AAGATGAATAA**GCTAGC**TCTAGAGGATC	76			16237			
		GVR	GATCCTCTAGA**GCTAGC**TTATTCATCTT	794	1751	6.1871	9566	49631	10.4898	1.6954
	g034c	VGF	ATGCCTGCAG**GCGGCCGC**ATGGATAAGA	703			6452			
		VGR	TCTTATCCAT**GCGGCCGC**CTGCAGGCAT	3			6920			
		GVF	TATTAATATAA**GCTAGC**TCTAGAGGATC	441			8702			
		GVR	GATCCTCTAGA**GCTAGC**TTATATTAATA	891	2038	7.2012	5370	27444	5.8005	0.8055
	g039c	VGF	ATGCCTGCAG**GCGGCCGC**ATGGAAAAAA	1086			9984			
		VGR	TTTTTTCCAT**GCGGCCGC**CTGCAGGCAT	5			11647			
		GVF	AATTAACTTGA**GCTAGC**TCTAGAGGATC	55			15136			
		GVR	GATCCTCTAGA**GCTAGC**TCAAGTTAATT	1104	2250	7.9502	8761	45528	9.6226	1.2104

	g040c	VGF	ATGCCTGCAG**GCGGCCGC**ATGAAAAATA	296			7810			
		VGR	TATTTTTCAT**GCGGCCGC**CTGCAGGCAT	1			10260			
		GVF	TTTGGGAGTAA**GCTAGC**TCTAGAGGATC	99			12182			
		GVR	GATCCTCTAGA**GCTAGC**TTACTCCCAAA	653	1049	3.7066	7974	38226	8.0793	2.1797
	g042c	VGF	ATGCCTGCAG**GCGGCCGC**ATGAACTTAG	1160			7959			
		VGR	CTAAGTTCAT**GCGGCCGC**CTGCAGGCAT	3			9039			
		GVF	ATGAGAGATAA**GCTAGC**TCTAGAGGATC	43			11325			
		GVR	GATCCTCTAGA**GCTAGC**TTATCTCTCAT	1901	3107	10.9784	8065	36388	7.6908	0.7005
	g043c	VGF	ATGCCTGCAG**GCGGCCGC**ATGGACTTTT	353			5639			
		VGR	AAAAGTCCAT**GCGGCCGC**CTGCAGGCAT	3			6674			
		GVF	ATGGTTATTAA**GCTAGC**TCTAGAGGATC	117			8861			
		GVR	GATCCTCTAGA**GCTAGC**TTAATAACCAT	926	1399	4.9433	4945	26119	5.5204	1.1167
	g044c	VGF	ATGCCTGCAG**GCGGCCGC**ATGTTTAAAA	761			4666			
		VGR	TTTTAAACAT**GCGGCCGC**CTGCAGGCAT	4			5404			
		GVF	AGGATGAGTAA**GCTAGC**TCTAGAGGATC	100			6231			
		GVR	GATCCTCTAGA**GCTAGC**TTACTCATCCT	1044	1909	6.7453	5080	21381	4.5190	0.6699
	g045c	VGF	ATGCCTGCAG**GCGGCCGC**ATGGAAAAAG	79			7532			
		VGR	CTTTTTCCAT**GCGGCCGC**CTGCAGGCAT	3			8772			
		GVF	ATGCTAAATAA**GCTAGC**TCTAGAGGATC	71			10313			
		GVR	GATCCTCTAGA**GCTAGC**TTATTTAGCAT	323	476	1.6819	7217	33834	7.1510	4.2517
	g046c	VGF	ATGCCTGCAG**GCGGCCGC**ATGGCAAATG	619			4333			
		VGR	CATTTGCCAT**GCGGCCGC**CTGCAGGCAT	3			5196			
		GVF	AAGACCTATGA**GCTAGC**TCTAGAGGATC	716			5626			
		GVR	GATCCTCTAGA**GCTAGC**TCATAGGTCTT	728	2066	7.3001	4359	19514	4.1244	0.5650
	g053c	VGF	ATGCCTGCAG**GCGGCCGC**GTGTCTAAAA	864			5144			
		VGR	TTTTAGACAC**GCGGCCGC**CTGCAGGCAT	5			6568			
		GVF	TGTATTTTTAA**GCTAGC**TCTAGAGGATC	84			6527			
		GVR	GATCCTCTAGA**GCTAGC**TTAAAAATACA	1399	2352	8.3107	4628	22867	4.8331	0.5816
	g056c	VGF	ATGCCTGCAG**GCGGCCGC**ATGGAAAAAT	150			7190			
		VGR	ATTTTTCCAT**GCGGCCGC**CTGCAGGCAT	2			9591			
		GVF	ATATAGAATAA**GCTAGC**TCTAGAGGATC	174			10619			
		GVR	GATCCTCTAGA**GCTAGC**TTATTCTATAT	400	726	2.5653	6410	33810	7.1460	2.7856
	Total			28301			473135			
3	*g061c*	VGF	ATGCCTGCAG**GCGGCCGC**ATGGATAATT	1559			14144			
		VGR	AATTATCCAT**GCGGCCGC**CTGCAGGCAT	16	3121	14.0371	10584	48959	9.5298	0.6789

	GVF	CAAGGAGGTAG**GCTAGC**TCTAGAGGATC	208			12048			
	GVR	GATCCTCTAGA**GCTAGC**CTACCTCCTTG	1338			12183			
g062c	VGF	ATGCCTGCAG**GCGGCCGC**ATGGTAAATA	779			5			
	VGR	TATTTACCAT**GCGGCCGC**CTGCAGGCAT	1000			0			
	GVF	AAGATGGATAA**GGTACC**GAGCTCGAATT	0			2			
	GVR	AATTCGAGCTC**GGTACC**TTATCCATCTT	0	1779	8.0013	0	7	0.0014	0.0002
g065c	VGF	ATGCCTGCAG**GCGGCCGC**ATGAATTATT	412			14899			
	VGR	AATAATTCAT**GCGGCCGC**CTGCAGGCAT	4			14530			
	GVF	AGGATAATTAA**GCTAGC**TCTAGAGGATC	81			15130			
	GVR	GATCCTCTAGA**GCTAGC**TTAATTATCCT	654	1151	5.1768	13964	58523	11.3914	2.2005
g069c	VGF	ATGCCTGCAG**GCGGCCGC**ATGAAAAAAG	126			10685			
	VGR	CTTTTTTCAT**GCGGCCGC**CTGCAGGCAT	12			11462			
	GVF	ATGCAGGATAG**GCTAGC**TCTAGAGGATC	60			12853			
	GVR	GATCCTCTAGA**GCTAGC**CTATCCTGCAT	334	532	2.3927	10922	45922	8.9386	3.7357
g072c	VGF	ATGCCTGCAG**GCGGCCGC**ATGAATAAAT	400			9677			
	VGR	ATTTATTCAT**GCGGCCGC**CTGCAGGCAT	15			9153			
	GVF	AGGAGAAATAA**GCTAGC**TCTAGAGGATC	611			10772			
	GVR	GATCCTCTAGA**GCTAGC**TTATTTCTCCT	440	1466	6.5935	9529	39131	7.6168	1.1552
g075c	VGF	ATGCCTGCAG**GCGGCCGC**ATGGCACTAC	494			7644			
	VGR	GTAGTGCCAT**GCGGCCGC**CTGCAGGCAT	5			6296			
	GVF	GAGAAATGTAA**GCTAGC**TCTAGAGGATC	226	27		7114			
	GVR	GATCCTCTAGA**GCTAGC**TTACATTTCTC	1984		12.1840	6379	27433	5.3398	0.4383
g078c	VGF	ATGCCTGCAG**GCGGCCGC**ATGGCATCAG	45			2562			
	VGR	CTGATGCCAT**GCGGCCGC**CTGCAGGCAT	0			2299			
	GVF	TTAATGAGTAA**GCTAGC**TCTAGAGGATC	160	4		2929			
	GVR	GATCCTCTAGA**GCTAGC**TTACTCATTAA	276		2.1634	2319	10109	1.9677	0.9096
g079c	VGF	ATGCCTGCAG**GCGGCCGC**ATGAAAAGAC	128			3063			
	VGR	GTCTTTTCAT**GCGGCCGC**CTGCAGGCAT	2			2689			
	GVF	AAGATAACTAA**GCTAGC**TCTAGAGGATC	60			3836			
	GVR	GATCCTCTAGA**GCTAGC**TTAGTTATCTT	572	2	3.4272	3156	12744	2.4806	0.7238
g080c	VGF	ATGCCTGCAG**GCGGCCGC**ATGTCAAAAC	64			4665			
	VGR	GTTTTGACAT**GCGGCCGC**CTGCAGGCAT	2			4205			
	GVF	TAGTATTCTAA**GCTAGC**TCTAGAGGATC	48			4954			
	GVR	GATCCTCTAGA**GCTAGC**TTAGAATACTA	358	72	2.1229	4167	17991	3.5019	1.6496
g081c	VGF	ATGCCTGCAG**GCGGCCGC**TTGGATAAGG	349	1324	5.9548	42	152	0.0296	0.0050

		VGR	CCTTATCCAA**GCGGCCGC**CTGCAGGCAT	2			35			
		GVF	AATTTGCATAG**GCTAGC**TCTAGAGGATC	45			40			
		GVR	GATCCTCTAGA**GCTAGC**CTATGCAAATT	928			35			
	g083c	VGF	ATGCCTGCAG**GCGGCCGC**ATGATTATCG	196			5441			
		VGR	CGATAATCAT**GCGGCCGC**CTGCAGGCAT	2			4797			
		GVF	AAAATAAGTAA**GCTAGC**TCTAGAGGATC	48			4993			
		GVR	GATCCTCTAGA**GCTAGC**TTACTTATTTT	1088	1334	5.9998	4839	20070	3.9066	0.6511
	g085	VGF	ATGCCTGCAG**GCGGCCGC**ATGAAAACAA	320			1			
		VGR	TTGTTTTCAT**GCGGCCGC**CTGCAGGCAT	39			1			
		GVF	AAATGGATTGA**GGTACC**GAGCTCGAATT	0			0			
		GVR	AATTCGAGCTC**GGTACC**TCAATCCATTT	0	359	1.6146	0	2	0.0004	0.0002
	g086	VGF	ATGCCTGCAG**GCGGCCGC**TTGAGTGCAG	787			15794			
		VGR	CTGCACTCAA**GCGGCCGC**CTGCAGGCAT	20			11996			
		GVF	AAACATTCTAA**GCTAGC**TCTAGAGGATC	107			16324			
		GVR	GATCCTCTAGA**GCTAGC**TTAGAATGTTT	1068	1982	8.9143	16931	61045	11.8823	1.3329
	g089	VGF	ATGCCTGCAG**GCGGCCGC**GTGATTTTAT	91			16926			
		VGR	ATAAAATCAC**GCGGCCGC**CTGCAGGCAT	2			16141			
		GVF	GAAATAAATGA**GCTAGC**TCTAGAGGATC	118			19076			
		GVR	GATCCTCTAGA**GCTAGC**TCATTTATTTC	232	443	1.9924	15550	67693	13.1763	6.6131
	g092	VGF	ATGCCTGCAG**GCGGCCGC**ATGAATATTA	1042			15222			
		VGR	TAATATTCAT**GCGGCCGC**CTGCAGGCAT	8			11823			
		GVF	ATAAAAAATAA**GCTAGC**TCTAGAGGATC	120			12184			
		GVR	GATCCTCTAGA**GCTAGC**TTATTTTTTAT	1648	2818	12.6743	11679	50908	9.9091	0.7818
	g093	VGF	ATGCCTGCAG**GCGGCCGC**TTGATATTCT	575			13067			
		VGR	AGAATATCAA**GCGGCCGC**CTGCAGGCAT	0			11160			
		GVF	GAGGTGACTAG**GCTAGC**TCTAGAGGATC	50			14443			
		GVR	GATCCTCTAGA**GCTAGC**CTAGTCACCTC	876	1501	6.7509	14390	53060	10.3280	1.5299
	Total			22234			513749			
4	*g103*	VGF	ATGCCTGCAG**GCGGCCGC**ATGGCTAGAA	295			14649			
		VGR	TTCTAGCCAT**GCGGCCGC**CTGCAGGCAT	3			14742			
		GVF	TAGATATATAA**GCTAGC**TCTAGAGGATC	136			14589			
		GVR	GATCCTCTAGA**GCTAGC**TTATATATCTA	578	1012	2.5216	13232	57212	6.7445	2.6747
	g107	VGF	ATGCCTGCAG**GCGGCCGC**ATGAGTACAT	423			5214			
		VGR	ATGTACTCAT**GCGGCCGC**CTGCAGGCAT	6			4472			
		GVF	CAATTCAATAA**GCTAGC**TCTAGAGGATC	25	1185	2.9527	4733	19181	2.2612	0.7658

	GVR	GATCCTCTAGA**GCTAGC**TTATTGAATTG	731			4762			
g108	VGF	ATGCCTGCAG**GCGGCCGC**ATGGGTATAA	196			10591			
	VGR	TTATACCCAT**GCGGCCGC**CTGCAGGCAT	1			11567			
	GVF	AATTTATGTAG**GCTAGC**TCTAGAGGATC	37			12880			
	GVR	GATCCTCTAGA**GCTAGC**CTACATAAATT	282	516	1.2857	10029	45067	5.3128	4.1321
g109	VGF	ATGCCTGCAG**GCGGCCGC**ATGTCACAAG	1462			11628			
	VGR	CTTGTGACAT**GCGGCCGC**CTGCAGGCAT	8			9564			
	GVF	ATGTAAAGTAA**GCTAGC**TCTAGAGGATC	70			12109			
	GVR	GATCCTCTAGA**GCTAGC**TTACTTTACAT	1883	3423	8.5291	10600	43901	5.1753	0.6068
g131	VGF	ATGCCTGCAG**GCGGCCGC**ATGAAAAAAT	2010			19918			
	VGR	ATTTTTTCAT**GCGGCCGC**CTGCAGGCAT	19			17931			
	GVF	GGGATAAGTAA**GCTAGC**TCTAGAGGATC	88			20080			
	GVR	GATCCTCTAGA**GCTAGC**TTACTTATCCC	1687	3804	9.4785	19345	77274	9.1095	0.9611
g135	VGF	ATGCCTGCAG**GCGGCCGC**ATGTCAAATA	2214			21754			
	VGR	TATTTGACAT**GCGGCCGC**CTGCAGGCAT	9			17892			
	GVF	AACAAGAATAA**GCTAGC**TCTAGAGGATC	211			23892			
	GVR	GATCCTCTAGA**GCTAGC**TTATTCTTGTT	2045	4479	11.1604	17129	80667	9.5095	0.8521
g136	VGF	ATGCCTGCAG**GCGGCCGC**ATGGAAAAAA	1044			10515			
	VGR	TTTTTTCCAT**GCGGCCGC**CTGCAGGCAT	2			8105			
	GVF	ACGAACAGTAA**GCTAGC**TCTAGAGGATC	114			11642			
	GVR	GATCCTCTAGA**GCTAGC**TTACTGTTCGT	1294	2454	6.1147	9623	39885	4.7019	0.7690
g141	VGF	ATGCCTGCAG**GCGGCCGC**ATGGATAGAA	1840			17450			
	VGR	TTCTATCCAT**GCGGCCGC**CTGCAGGCAT	9			16618			
	GVF	AAAATGAATAG**GCTAGC**TCTAGAGGATC	81			18377			
	GVR	GATCCTCTAGA**GCTAGC**CTATTCATTTT	1621	3551	8.8481	16339	68784	8.1087	0.9164
g146	VGF	ATGCCTGCAG**GCGGCCGC**GTGAATACGG	764			22415			
	VGR	CCGTATTCAC**GCGGCCGC**CTGCAGGCAT	7			21605			
	GVF	TTAATATTTAA**GCTAGC**TCTAGAGGATC	75			22639			
	GVR	GATCCTCTAGA**GCTAGC**TTAAATATTAA	877	1723	4.2932	19599	86258	10.1686	2.3685
g150	VGF	ATGCCTGCAG**GCGGCCGC**ATGGATAATT	2145			16698			
	VGR	AATTATCCAT**GCGGCCGC**CTGCAGGCAT	4			14616			
	GVF	AAGAAAGCTAA**GCTAGC**TCTAGAGGATC	56			18003			
	GVR	GATCCTCTAGA**GCTAGC**TTAGCTTTCTT	2049	4254	10.5998	15909	65226	7.6893	0.7254
g155	VGF	ATGCCTGCAG**GCGGCCGC**ATGGATATTC	806			12255			
	VGR	GAATATCCAT**GCGGCCGC**CTGCAGGCAT	8	1889	4.7068	11693	48382	5.7036	1.2118

		GVF	TAGGTGAGTAG**GCTAGC**TCTAGAGGATC	122			12197			
		GVR	GATCCTCTAGA**GCTAGC**CTACTCACCTA	953			12237			
	g156	VGF	ATGCCTGCAG**GCGGCCGC**ATGGGAATTA	1958			5361			
		VGR	TAATTCCCAT**GCGGCCGC**CTGCAGGCAT	6			4467			
		GVF	GTTATGAGTAA**GCTAGC**TCTAGAGGATC	78			5644			
		GVR	GATCCTCTAGA**GCTAGC**TTACTCATAAC	2134	4176	10.4054	4467	19939	2.3505	0.2259
	g159	VGF	ATGCCTGCAG**GCGGCCGC**ATGAAGTTCA	1799			5820			
		VGR	TGAACTTCAT**GCGGCCGC**CTGCAGGCAT	10			5161			
		GVF	ATTTCTTATAG**GCTAGC**TCTAGAGGATC	49			5817			
		GVR	GATCCTCTAGA**GCTAGC**CTATAAGAAAT	1609	3467	8.6388	5529	22327	2.6320	0.3047
	g166	VGF	ATGCCTGCAG**GCGGCCGC**ATGTTTATTT	1205			14716			
		VGR	AAATAAACAT**GCGGCCGC**CTGCAGGCAT	3			13389			
		GVF	TCATTGAGTAA**GCTAGC**TCTAGAGGATC	68			16807			
		GVR	GATCCTCTAGA**GCTAGC**TTACTCAATGA	1201	2477	6.1720	13822	58734	6.9239	1.1218
	g169	VGF	ATGCCTGCAG**GCGGCCGC**ATGGAAATGG	522			13832			
		VGR	CCATTTCCAT**GCGGCCGC**CTGCAGGCAT	8			12031			
		GVF	AAAGGAGGTAG**GCTAGC**TCTAGAGGATC	85			15266			
		GVR	GATCCTCTAGA**GCTAGC**CTACCTCCTTT	545	1160	2.8904	12422	53551	6.3129	2.1841
	g171	VGF	ATGCCTGCAG**GCGGCCGC**ATGGTTATAC	141			14296			
		VGR	GTATAACCAT**GCGGCCGC**CTGCAGGCAT	7			15531			
		GVF	ATGGTGAGTAA**GCTAGC**TCTAGAGGATC	127			18288			
		GVR	GATCCTCTAGA**GCTAGC**TTACTCACCAT	288	563	1.4028	13772	61887	7.2956	5.2006
	Total			40133			848275			
5	*g172*	VGF	ATGCCTGCAG**GCGGCCGC**ATGGTGAGTA	2563			3951			
		VGR	TACTCACCAT**GCGGCCGC**CTGCAGGCAT	1953			3861			
		GVF	AAAATGAATAA**GGTACC**GAGCTCGAATT	1872			4162			
		GVR	AATTCGAGCTC**GGTACC**TTATTCATTTT	1876	8264	15.4612	3654	15628	2.4312	0.1572
	g173	VGF	ATGCCTGCAG**GCGGCCGC**ATGAATAAAG	430			9036			
		VGR	CTTTATTCAT**GCGGCCGC**CTGCAGGCAT	3			9107			
		GVF	AACCAGGCTAA**GCTAGC**TCTAGAGGATC	108			10610			
		GVR	GATCCTCTAGA**GCTAGC**TTAGCCTGGTT	791	1332	2.4920	9070	37823	5.8840	2.3611
	g175	VGF	ATGCCTGCAG**GCGGCCGC**ATGATAAGCT	2655			1577			
		VGR	AGCTTATCAT**GCGGCCGC**CTGCAGGCAT	1501			1506			
		GVF	TTTTACTATAG**GGTACC**GAGCTCGAATT	1504			1581			
		GVR	AATTCGAGCTC**GGTACC**CTATAGTAAAA	1505	7165	13.4051	1287	5951	0.9258	0.0691

g176	VGF	ATGCCTGCAG**GCGGCCGC**ATGGATTTTA	203			13572			
	VGR	TAAAATCCAT**GCGGCCGC**CTGCAGGCAT	13			13653			
	GVF	GAAATGACTAA**GCTAGC**TCTAGAGGATC	142			19780			
	GVR	GATCCTCTAGA**GCTAGC**TTAGTCATTTC	310	668	1.2498	11411	58416	9.0876	7.2714
g177	VGF	ATGCCTGCAG**GCGGCCGC**ATGACTAAAG	939			5024			
	VGR	CTTTAGTCAT**GCGGCCGC**CTGCAGGCAT	3			4536			
	GVF	AAGCATTTTAA**GCTAGC**TCTAGAGGATC	48			4907			
	GVR	GATCCTCTAGA**GCTAGC**TTAAAATGCTT	1486	2476	4.6324	4815	19282	2.9996	0.6475
g179	VGF	ATGCCTGCAG**GCGGCCGC**ATGGATATAC	2048			16889			
	VGR	GTATATCCAT**GCGGCCGC**CTGCAGGCAT	7			13147			
	GVF	TAATGTTATAA**GCTAGC**TCTAGAGGATC	75			15998			
	GVR	GATCCTCTAGA**GCTAGC**TTATAACATTA	1832	3962	7.4125	14793	60827	9.4626	1.2766
g181	VGF	ATGCCTGCAG**GCGGCCGC**ATGCCTATGG	972			8760			
	VGR	CCATAGGCAT**GCGGCCGC**CTGCAGGCAT	7			7904			
	GVF	CATTTTCTTAA**GCTAGC**TCTAGAGGATC	52			8649			
	GVR	GATCCTCTAGA**GCTAGC**TTAAGAAAATG	1386	2417	4.5220	7246	32559	5.0651	1.1201
g182	VGF	ATGCCTGCAG**GCGGCCGC**ATGATTAATA	1575			11392			
	VGR	TATTAATCAT**GCGGCCGC**CTGCAGGCAT	10			8969			
	GVF	TACAATTATAG**GCTAGC**TCTAGAGGATC	54			12104			
	GVR	GATCCTCTAGA**GCTAGC**CTATAATTGTA	1691	3330	6.2301	9725	42190	6.5633	1.0535
g183	VGF	ATGCCTGCAG**GCGGCCGC**ATGAGAGAAG	2133			16463			
	VGR	CTTCTCTCAT**GCGGCCGC**CTGCAGGCAT	11			14642			
	GVF	AGGAAAAATAA**GCTAGC**TCTAGAGGATC	187			16520			
	GVR	GATCCTCTAGA**GCTAGC**TTATTTTTCCT	2114	4445	8.3162	15280	62905	9.7859	1.1767
g185	VGF	ATGCCTGCAG**GCGGCCGC**ATGAATGAGT	161			12612			
	VGR	ACTCATTCAT**GCGGCCGC**CTGCAGGCAT	7			13467			
	GVF	AGGAGAGATAA**GCTAGC**TCTAGAGGATC	205			18012			
	GVR	GATCCTCTAGA**GCTAGC**TTATCTCTCCT	344	717	1.3414	11823	55914	8.6983	6.4843
g187	VGF	ATGCCTGCAG**GCGGCCGC**ATGAAGCAGA	1979			2574			
	VGR	TCTGCTTCAT**GCGGCCGC**CTGCAGGCAT	27			1955			
	GVF	TAGATATTTAA**GCTAGC**TCTAGAGGATC	130			2518			
	GVR	GATCCTCTAGA**GCTAGC**TTAAATATCTA	2000	4136	7.7381	1748	8795	1.3682	0.1768
g188	VGF	ATGCCTGCAG**GCGGCCGC**ATGAACAAAG	1783			14223			
	VGR	CTTTGTTCAT**GCGGCCGC**CTGCAGGCAT	11			11812			
	GVF	TTATAAAGTAG**GCTAGC**TCTAGAGGATC	77	3433	6.4228	12860	50504	7.8567	1.2232

		GVR	GATCCTCTAGA**GCTAGC**CTACTTTATAA	1562			11609			
	g190	VGF	ATGCCTGCAG**GCGGCCGC**ATGAATGGTA	1893			8627			
		VGR	TACCATTCAT**GCGGCCGC**CTGCAGGCAT	14			7671			
		GVF	TGAGTCAATAA**GCTAGC**TCTAGAGGATC	144			8981			
		GVR	GATCCTCTAGA**GCTAGC**TTATTGACTCA	1927	3978	7.4425	8067	33346	5.1875	0.6970
	g191	VGF	ATGCCTGCAG**GCGGCCGC**ATGGTAATTG	1389			15268			
		VGR	CAATTACCAT**GCGGCCGC**CTGCAGGCAT	11			13432			
		GVF	ATAAGGAGTGA**GCTAGC**TCTAGAGGATC	64			15746			
		GVR	GATCCTCTAGA**GCTAGC**TCACTCCTTAT	1271	2735	5.1169	14498	58944	9.1697	1.7920
	g192	VGF	ATGCCTGCAG**GCGGCCGC**ATGGAATTTA	649			10453			
		VGR	TAAATTCCAT**GCGGCCGC**CTGCAGGCAT	11			10588			
		GVF	ACATACTATGA**GCTAGC**TCTAGAGGATC	66			12407			
		GVR	GATCCTCTAGA**GCTAGC**TCATAGTATGT	756	1482	2.7727	10773	44221	6.8793	2.4811
	g194	VGF	ATGCCTGCAG**GCGGCCGC**GTGGAGAAAT	1498			14979			
		VGR	ATTTCTCCAC**GCGGCCGC**CTGCAGGCAT	4			12749			
		GVF	GAGGGAAATAA**GCTAGC**TCTAGAGGATC	112			16970			
		GVR	GATCCTCTAGA**GCTAGC**TTATTTCCCTC	1296	2910	5.4443	10810	55508	8.6352	1.5861
	Total			53450			642813			
6	*g195*	VGF	ATGCCTGCAG**GCGGCCGC**ATGGAATATT	576			21518			
		VGR	AATATTCCAT**GCGGCCGC**CTGCAGGCAT	3			21476			
		GVF	TATTCTTTTAA**GCTAGC**TCTAGAGGATC	55			24508			
		GVR	GATCCTCTAGA**GCTAGC**TTAAAAGAATA	634	1268	4.7775	20210	87712	13.9325	2.9163
	g196	VGF	ATGCCTGCAG**GCGGCCGC**ATGAAACATT	113			22625			
		VGR	AATGTTTCAT**GCGGCCGC**CTGCAGGCAT	2			26451			
		GVF	GTAGAAATTAA**GCTAGC**TCTAGAGGATC	84			28687			
		GVR	GATCCTCTAGA**GCTAGC**TTAATTTCTAC	297	496	1.8688	22396	100159	15.9096	8.5133
	g198	VGF	ATGCCTGCAG**GCGGCCGC**ATGAAAGTAG	1272			16580			
		VGR	CTACTTTCAT**GCGGCCGC**CTGCAGGCAT	9			14317			
		GVF	AGGAAAAATAA**GCTAGC**TCTAGAGGATC	34			15988			
		GVR	GATCCTCTAGA**GCTAGC**TTATTTTTCCT	918	2233	8.4134	14888	61773	9.8123	1.1663
	g199	VGF	ATGCCTGCAG**GCGGCCGC**GTGTCTAATA	1585			31107			
		VGR	TATTAGACAC**GCGGCCGC**CTGCAGGCAT	3			27280			
		GVF	TTAAAAATTAA**GCTAGC**TCTAGAGGATC	51			28325			
		GVR	GATCCTCTAGA**GCTAGC**TTAATTTTTAA	2520	4159	15.6701	30016	116728	18.5415	1.1832
	g201	VGF	ATGCCTGCAG**GCGGCCGC**ATGAATTATG	129	570	2.1476	5882	24692	3.9222	1.8263

	VGR	CATAATTCAT**GCGGCCGC**CTGCAGGCAT	1			5945			
	GVF	TTTTATTTTAA**GCTAGC**TCTAGAGGATC	40			7118			
	GVR	GATCCTCTAGA**GCTAGC**TTAAAATAAAA	400			5747			
g202	VGF	ATGCCTGCAG**GCGGCCGC**ATGAATTATA	98			2961			
	VGR	TATAATTCAT**GCGGCCGC**CTGCAGGCAT	1			3102			
	GVF	AGGGGTTATAA**GCTAGC**TCTAGAGGATC	119			3790			
	GVR	GATCCTCTAGA**GCTAGC**TTATAACCCCT	574	792	2.9841	2962	12815	2.0356	0.6822
g204	VGF	ATGCCTGCAG**GCGGCCGC**ATGTATCCTG	608			9444			
	VGR	CAGGATACAT**GCGGCCGC**CTGCAGGCAT	1			7892			
	GVF	CAACAAAATGA**GCTAGC**TCTAGAGGATC	78			9925			
	GVR	GATCCTCTAGA**GCTAGC**TCATTTTGTTG	1376	2063	7.7729	8468	35729	5.6753	0.7301
g206	VGF	ATGCCTGCAG**GCGGCCGC**ATGGTAAAAT	797			13275			
	VGR	ATTTTACCAT**GCGGCCGC**CTGCAGGCAT	5			11383			
	GVF	GAGAATACTAA**GCTAGC**TCTAGAGGATC	137			13683			
	GVR	GATCCTCTAGA**GCTAGC**TTAGTATTCTC	1403	2342	8.8241	12359	50700	8.0534	0.9127
g208	VGF	ATGCCTGCAG**GCGGCCGC**ATGATTTATA	157			4288			
	VGR	TATAAATCAT**GCGGCCGC**CTGCAGGCAT	1			4228			
	GVF	TACAGCCATAG**GCTAGC**TCTAGAGGATC	48			5572			
	GVR	GATCCTCTAGA**GCTAGC**CTATGGCTGTA	523	729	2.7467	4290	18378	2.9192	1.0628
g209	VGF	ATGCCTGCAG**GCGGCCGC**ATGATTATAG	269			3734			
	VGR	CTATAATCAT**GCGGCCGC**CTGCAGGCAT	4			2978			
	GVF	GAGAGAAATAG**GCTAGC**TCTAGAGGATC	652			4115			
	GVR	GATCCTCTAGA**GCTAGC**CTATTTCTCTC	620	1545	5.8212	3646	14473	2.2989	0.3949
g210	VGF	ATGCCTGCAG**GCGGCCGC**ATGAGTAATA	56			5881			
	VGR	TATTACTCAT**GCGGCCGC**CTGCAGGCAT	0			5983			
	GVF	CAGATAAATAA**GCTAGC**TCTAGAGGATC	183			6909			
	GVR	GATCCTCTAGA**GCTAGC**TTATTTATCTG	388	627	2.3624	5843	24616	3.9101	1.6552
g211	VGF	ATGCCTGCAG**GCGGCCGC**ATGATGAATG	493			7520			
	VGR	CATTCATCAT**GCGGCCGC**CTGCAGGCAT	0			6642			
	GVF	AAGGTATGTAA**GCTAGC**TCTAGAGGATC	87			8579			
	GVR	GATCCTCTAGA**GCTAGC**TTACATACCTT	1219	1799	6.7782	7327	30068	4.7761	0.7046
g212	VGF	ATGCCTGCAG**GCGGCCGC**ATGAAAAAAC	509			2261			
	VGR	GTTTTTTCAT**GCGGCCGC**CTGCAGGCAT	62			1753			
	GVF	AAGGAGATTAA**GCTAGC**TCTAGAGGATC	221			2100			
	GVR	GATCCTCTAGA**GCTAGC**TTAATCTCCTT	996	1788	6.7367	1831	7945	1.2620	0.1873

g213	VGF	ATGCCTGCAG**GCGGCCGC**ATGTATATAT	974			6394			
	VGR	ATATATACAT**GCGGCCGC**CTGCAGGCAT	179			6264			
	GVF	ATGACTTATGA**GCTAGC**TCTAGAGGATC	254			8408			
	GVR	GATCCTCTAGA**GCTAGC**TCATAAGTCAT	1562	2969	11.1865	6269	27335	4.3420	0.3881
g215	VGF	ATGCCTGCAG**GCGGCCGC**ATGATAAATA	216			2012			
	VGR	TATTTATCAT**GCGGCCGC**CTGCAGGCAT	68			1869			
	GVF	AACGATGGTAA**GCTAGC**TCTAGAGGATC	125			2756			
	GVR	GATCCTCTAGA**GCTAGC**TTACCATCGTT	755	1164	4.3857	1857	8494	1.3492	0.3076
g216	VGF	ATGCCTGCAG**GCGGCCGC**ATGAATGCTA	589			2226			
	VGR	TAGCATTCAT**GCGGCCGC**CTGCAGGCAT	64			1729			
	GVF	TTAGTTGGTAA**GCTAGC**TCTAGAGGATC	265			2040			
	GVR	GATCCTCTAGA**GCTAGC**TTACCAACTAA	1079	1997	7.5242	1937	7932	1.2599	0.1675
Total			26541			629549			
