# Supplementary material for: Discovery of Bactericidal Proteins from Staphylococcus Phage Stab21 Using a High-Throughput Screening Method
Source: Antibiotics (Basel). 2023 Jul 21;12(7):1213. doi: 10.3390/antibiotics12071213 (PMC10376165; doi:10.3390/antibiotics12071213)
Supplement: Supplementary file 1 [file antibiotics-12-01213-s001.zip › Table S2.pdf]

Table S2. Recombinant strains used in this project. Cm30, chloramphenicol 30 μg/mL; Amp100, ampicillin 100 μg/mL. LB, lysogeny broth; LA, LB supplemented with 1.5% Bacto agar; TSA, tryptic soy agar; TSB, tryptic soy broth; “, same as previous line.

Host/strain number	Plasmid	Growing condition/usage
E. coli DH5α		
7213	pBAD33-g008	LA/LB, Cm30
7214	pBAD33-g024	“
7215	pBAD33-g062c	“
7216	pBAD33-g075c	“
7217	pBAD33-g081c	“
7218	pBAD33-g085	“
7219	pBAD33-g156	“
7220	pBAD33-g159	“
7221	pBAD33-g172	“
7222	pBAD33-g175	“
7223	pBAD33-g187	“
7224	pBAD33-g209	“
7225	pBAD33-g212	“
7226	pBAD33-g213	“
7227	pBAD33-g215	“
7228	pBAD33-g216	“
7298	pRAB11N-ORF104	Toxic control
E. coli DH10B		
7249	pRAB11N	TSA/TSB, Amp100
7242	pRAB11N-g024	“
7243	pRAB11N-g081c	“
7244	pRAB11N-g159	“
7245	pRAB11N-g172	“
7246	pRAB11N-g175	“
7247	pRAB11N-g187	“
7248	pRAB11N-g209	“
7264	pRAB11N-g212	“
7265	pRAB11N-g213	“
S. aureus RN4220		
6497	plasmid-free	TSA/TSB
7266	pRAB11N	TSA/TSB, Cm30
7267	pRAB11N-g024	“
7268	pRAB11N-g081c	“
7269	pRAB11N-g159	“
7270	pRAB11N-g172	“
7271	pRAB11N-g175	“
7272	pRAB11N-g187	“
7273	pRAB11N-g209	“
7274	pRAB11N-g212	“
7275	pRAB11N-g213	“
7297	pRAB11N-ORF104	Toxic control
