# Supplementary material for: Discovery of Bactericidal Proteins from Staphylococcus Phage Stab21 Using a High-Throughput Screening Method
Source: Antibiotics (Basel). 2023 Jul 21;12(7):1213. doi: 10.3390/antibiotics12071213 (PMC10376165; doi:10.3390/antibiotics12071213)
Supplement: Supplementary file 1 [file antibiotics-12-01213-s001.zip › Table S3.pdf]

Table S3. Primers and oligonucleotides used in this study. Restriction sites used in this study are marked with underscores and specified.

Gene	Primer	Sequence	Restriction site	Size (bp)
Primers for pCU1LK construction.				
NOTdown		CCGGGGATCCTCTAGAGCTAGCTCCATGGTGGGCGGCCGCCTGCA	NheI, NotI	
NOTup		GGCGGCCGCCCACCATGGAGCTAGCTCTAGAGGATCCCCGGGTAC	NotI, NheI	
Primers for the confirmation of the construction of and correct gene insertions in pCU1LK				
Puc19-F		GTCGTGCCAGCTGCAGATCTGAATCGGCCAACGC		717
Puc19-R		TTCAGCAGAGCTCAGATACCAAAT		
Primer pairs for cloning 96 Stab21 HPUFs into pCU1LK.				
Stab002	F	GCAGCGGCCGCATGATAATAATACTATTTACGCAG	NotI	306
	R	GGTGCTAGCCTATAAGAATTTCTCTATATGTTCC	NheI	
Stab003	F	GCAGCGGCCGCATGATTGATATATACTTAGGAG	NotI	297
	R	GGTGCTAGCTTAAAATATCTCTTCTATTATTCTT	NheI	
Stab005	F	GCAGCGGCCGCATGATAGAAATTAGGTTAGACG	NotI	318
	R	GGTGCTAGCCTAATAATCTAAGTCAAAAGGGT	NheI	
Stab006	F	GCAGCGGCCGCATGATAGAGATATACCTTAGTG	NotI	291
	R	GGTGCTAGCTTACATCTCCTTTACATACTC	NheI	
Stab008	F	GCAGCGGCCGCATGGTTACTTTAACATACACTATT	NotI	237
	R	GGTGGTACCCTATCCTACGTGCCAAGC	KpnI	
Stab009	F	GCAGCGGCCGCATGATAGTTATATATACAGATGTTT	NotI	345
	R	GGTGCTAGCTCAATCCCCGCCCATAC	NheI	
Stab010c	F	GCAGCGGCCGCATGAAAATAAACTATATTCCAA	NotI	336
	R	GGTGCTAGCTTATAGAATATTTATAACATTGTATT	NheI	
Stab012	F	GCAGCGGCCGCATGACAAACAAAAATTACTTATAC	NotI	285
	R	GGTGCTAGCTTAATTCTTAACCGCTTCTATT	NheI	
Stab013	F	GCAGCGGCCGCATGATATTAGAAATAGAAACTAA	NotI	270
	R	GGTGCTAGCTTATTTAGTTTTTAATTCTACATTA	NheI	
Stab017	F	GCAGCGGCCGCATGAAAATGTTCAAATTACAAAA	NotI	354
	R	GGTGCTAGCTCAATGTCTGATTGGTCT	NheI	
Stab018	F	GCAGCGGCCGCATGAACAGATTAGAAATAGTAAA	NotI	219
	R	GGTGCTAGCTTATGCGTATTCTTCGATT	NheI	
Stab020	F	GCAGCGGCCGCATGATTACAATGACAAAAACAA	NotI	171
	R	GGTGCTAGCTTAAACAGTTTCTGAGTTCTT	NheI	
Stab021	F	GCAGCGGCCGCATGACAAATACAATACAAGCAT	NotI	468
	R	GGTGCTAGCCTACAGTGCCATTTTTTGC	NheI	
Stab022	F	GCAGCGGCCGCATGTTGAAGATGAATAAATAC	NotI	195
	R	GGTGCTAGCTTACATTTCTTCTACTACATAAC	NheI	
Stab024	F	GCAGCGGCCGCATGAACATTAACGAATATATAGG	NotI	654
	R	GGTGCTAGCCTACCTCCCTAAGTCTTTT	NheI	
Stab026	F	GCAGCGGCCGCATGATGAACATGACAAACT	NotI	186
	R	GGTGCTAGCTTAAAATATTCCATTTTGTTTTTTT	NheI	
Stab027c	F	GCAGCGGCCGCATGAAACTATACCAAGTAGAA	NotI	234
	R	GGTGCTAGCTTAGGTATTATTAACAACCTCT	NheI	
Stab028c	F	GCAGCGGCCGCATGAACAAAGAACAAGCC	NotI	483
	R	GGTGCTAGCTTACTTATTCTCCTTGATTTTTTTT	NheI	
Stab029c	F	GCAGCGGCCGCATGGGATTAGACTTTGAAG	NotI	405
	R	GGTGCTAGCTTATACATTTACACTCATGATTAA	NheI	

Stab030c	F	GCAGCGGCCGCATGGAAAATTATAAAAACTTTATT	NotI	429
	R	GGTGCTAGCTTATTTTTCCTCCTCTTCAT	NheI	
Stab031c	F	GCAGCGGCCGCATGAGATATGATATTAATGAAAAAT	NotI	246
	R	GGTGCTAGCTCATTGTGATTCCTCCTTA	NheI	
Stab033c	F	GCAGCGGCCGCATGAATATCAAATATATTGATTTAG	NotI	429
	R	GGTGCTAGCTTATTCATCTTCTTCCTCC	NheI	
Stab034c	F	GCAGCGGCCGCATGGATAAGATAAATCTCAATA	NotI	540
	R	GGTGCTAGCTTATATTAATAATTCTTTCCATTCT	NheI	
Stab039c	F	GCAGCGGCCGCATGGAAAAAATTTATATATTAGAAG	NotI	546
	R	GGTGCTAGCTCAAGTTAATTTATCAATTGAAT	NheI	
Stab040c	F	GCAGCGGCCGCATGAAAAATATTATTAATTTTTTAGT	NotI	216
	R	GGTGCTAGCTTACTCCCAAATACCAATA	NheI	
Stab042c	F	GCAGCGGCCGCATGAACTTAGAAAAAAGTTTC	NotI	735
	R	GGTGCTAGCTTATCTCTCATTATAGACCTC	NheI	
Stab043c	F	GCAGCGGCCGCATGGACTTTTACCAATTTC	NotI	237
	R	GGTGCTAGCTTAATAACCATGTTTAGTTACC	NheI	
Stab044c	F	GCAGCGGCCGCATGTTTAAAAAAGCACCTC	NotI	387
	R	GGTGCTAGCTTACTCATCCTTTTTAACGT	NheI	
Stab045c	F	GCAGCGGCCGCATGGAAAAAGTAAATCATGAG	NotI	171
	R	GGTGCTAGCTTATTTAGCATTGTATTTCCATT	NheI	
Stab046c	F	GCAGCGGCCGCATGGCAAATGAAAAAGAGA	NotI	480
	R	GGTGCTAGCTCATAGGTCTTTTTCTAAGTCA	NheI	
Stab053c	F	GCAGCGGCCGCGTGTCTAAAAGAACAGAC	NotI	324
	R	GGTGCTAGCTTAAAAATACATTAATTTAAAAAAATC	NheI	
Stab056c	F	GCAGCGGCCGCATGGAAAAATTCCAAGAAG	NotI	186
	R	GGTGCTAGCTTATTCTATATCTCCTTTAATTTCT	NheI	
Stab061c	F	GCAGCGGCCGCATGGATAATTTATCACATTACT	NotI	576
	R	GGTGCTAGCCTACCTCCTTGAGTAATAATT	NheI	
Stab062c	F	GCAGCGGCCGCATGGTAAATAAAATTAACGATAAA	NotI	621
	R	GGTGGTACCTTATCCATCTTGTTCCCC	KpnI	
Stab065c	F	GCAGCGGCCGCATGAATTATTTAGCTAAGGTAT	NotI	222
	R	GGTGCTAGCTTAATTATCCTCCTTTGAATTAT	NheI	
Stab069c	F	GCAGCGGCCGCATGAAAAAAGGAGTATTTACA	NotI	189
	R	GGTGCTAGCCTATCCTGCATACTTATAATCC	NheI	
Stab072c	F	GCAGCGGCCGCATGAATAAATTTAAAAGATGGTT	NotI	225
	R	GGTGCTAGCTTATTTCTCCTCTACTTTTAAAAA	NheI	
Stab075c	F	GCAGCGGCCGCATGGCACTACTTTTAACAT	NotI	306
	R	GGTGCTAGCTTACATTTCTCCTTTTTCTATTT	NheI	
Stab078c	F	GCAGCGGCCGCATGGCATCAGCAAAACAA	NotI	183
	R	GGTGCTAGCTTACTCATTAATTTGGTTTAGTTTTTT	NheI	
Stab079c	F	GCAGCGGCCGCATGAAAAGACAAAAAATGTTTT	NotI	216
	R	GGTGCTAGCTTAGTTATCTTTTGTTAATTCTTCC	NheI	
Stab080c	F	GCAGCGGCCGCATGTCAAAACATATTGAAATAA	NotI	207
	R	GGTGCTAGCTTAGAATACTATTTTAAAAGATTCT	NheI	
Stab081c	F	GCAGCGGCCGCTTGGATAAGGAGATAAACAAC	NotI	330
	R	GGTGCTAGCCTATGCAAATTTGTTAAAGACA	NheI	
Stab083c	F	GCAGCGGCCGCATGATTATCGTATCTTTTTTTCT	NotI	264
	R	GGTGCTAGCTTACTTATTTTGTGGTATAATAGTT	NheI	
Stab085	F	GCAGCGGCCGCATGAAAACAAAGAAAGAAATTAAA	NotI	276

	R	GGTGGTACCTCAATCCATTTCACCTCG	KpnI	
Stab086	F	GCAGCGGCCGCTTGAGTGCAGAAAATATTAGA	NotI	408
	R	GGTGCTAGCTTAGAATGTTTCTGAATTTTCC	NheI	
Stab089	F	GCAGCGGCCGCGTGATTTTATTTAGCACTATAATC	NotI	171
	R	GGTGCTAGCTCATTTATTTCTTCCTTCCTTT	NheI	
Stab092	F	GCAGCGGCCGCATGAATATTATAACGTCACTAT	NotI	339
	R	GGTGCTAGCTTATTTTTTATCTTTAAAGTTACTTT	NheI	
Stab093	F	GCAGCGGCCGCTTGATATTCTCTAAAGATAAAAAATG	NotI	369
	R	GGTGCTAGCCTAGTCACCTCTACTCCC	NheI	
Stab103	F	GCAGCGGCCGCATGGCTAGAAAAAAGACA	NotI	213
	R	GGTGCTAGCTTATATATCTAATTTCCTACCTAGA	NheI	
Stab107	F	GCAGCGGCCGCATGAGTACATTTTGGTCAG	NotI	456
	R	GGTGCTAGCTTATTGAATTGTCAAGTCTTTAC	NheI	
Stab108	F	GCAGCGGCCGCATGGGTATAACAATAGTAAATAG	NotI	192
	R	GGTGCTAGCCTACATAAATTTTAGTGACCAAT	NheI	
Stab109	F	GCAGCGGCCGCATGTCACAAGATAAATTAAGAG	NotI	309
	R	GGTGCTAGCTTACTTTACATATTCACCTGTAC	NheI	
Stab131	F	GCAGCGGCCGCATGAAAAAATATAGAGAATACCTA	NotI	375
	R	GGTGCTAGCTTACTTATCCCCTTTCGTAA	NheI	
Stab135	F	GCAGCGGCCGCATGTCAAATAATAAAAAAGATATTTT	NotI	336
	R	GGTGCTAGCTTATTCTTGTTCTCCTTTTTCTTCTTC	NheI	
Stab136	F	GCAGCGGCCGCATGGAAAAAAATATTAGCACA	NotI	450
	R	GGTGCTAGCTTACTGTTCGTCATTTTTCT	NheI	
Stab141	F	GCAGCGGCCGCATGGATAGAAAAGAAGCAAT	NotI	327
	R	GGTGCTAGCCTATTCATTTTTTCCATCTTCTG	NheI	
Stab146	F	GCAGCGGCCGCGTGAATACGGGAGAGATT	NotI	240
	R	GGTGCTAGCTTAAATATTAACTGAGATACTACTT	NheI	
Stab150	F	GCAGCGGCCGCATGGATAATTTAATAGATAAAAACA	NotI	351
	R	GGTGCTAGCTTAGCTTTCTTCATAAGGATT	NheI	
Stab155	F	GCAGCGGCCGCATGGATATTCCAACAATATTATTT	NotI	258
	R	GGTGCTAGCCTACTCACCTACTCTTTCAT	NheI	
Stab156	F	GCAGCGGCCGCATGGGAATTATAGTAAACTCC	NotI	753
	R	GGTGCTAGCTTACTCATAACTGCTTCCTT	NheI	
Stab159	F	GCAGCGGCCGCATGAAGTTCAATGATATTTATGA	NotI	309
	R	GGTGCTAGCCTATAAGAAATCCTTTTCCATTTTT	NheI	
Stab166	F	GCAGCGGCCGCATGTTTATTTCATTAAATCAAGAA	NotI	441
	R	GGTGCTAGCTTACTCAATGACAATACTATCC	NheI	
Stab169	F	GCAGCGGCCGCATGGAAATGGCAGATTTAG	NotI	240
	R	GGTGCTAGCCTACCTCCTTTGGTCTATTT	NheI	
Stab171	F	GCAGCGGCCGCATGGTTATACCTAGTATTAAAGC	NotI	174
	R	GGTGCTAGCTTACTCACCATATCTCTCCT	NheI	
Stab172	F	GCAGCGGCCGCATGGTGAGTAAATTTATCGG	NotI	249
	R	GGTGGTACCTTATTCATTTTCTTTATCCTTAATG	KpnI	
Stab173	F	GCAGCGGCCGCATGAATAAAGGGGAATTTATTAT	NotI	231
	R	GGTGCTAGCTTAGCCTGGTTGATTTACT	NheI	
Stab175	F	GCAGCGGCCGCATGATAAGCTCATTTGATAGT	NotI	246
	R	GGTGGTACCCTATAGTAAAATATTGTTTACTGCT	KpnI	
Stab176	F	GCAGCGGCCGCATGGATTTTAATGATTTTATAAACA	NotI	174
	R	GGTGCTAGCTTAGTCATTTCTTTTTCTCCTT	NheI	

Stab177	F	GCAGCGGCCGCATGACTAAAGAAACAAATGTAC	NotI	294
	R	GGTGCTAGCTTAAAATGCTTCATCTGTCAA	NheI	
Stab179	F	GCAGCGGCCGCATGGATATACTAATTATTCATTATAA	NotI	366
	R	GGTGCTAGCTTATAACATTAAGTCTTCATTTAAT	NheI	
Stab181	F	GCAGCGGCCGCATGCCTATGGACTTATTAAC	NotI	276
	R	GGTGCTAGCTTAAGAAAATGAAAGAAGATTTATT	NheI	
Stab182	F	GCAGCGGCCGCATGATTAATATGAGTAAAGAAAC	NotI	312
	R	GGTGCTAGCCTATAATTGTAACTTATGATAGTTAA	NheI	
Stab183	F	GCAGCGGCCGCATGAGAGAAGAGTTAAAACC	NotI	348
	R	GGTGCTAGCTTATTTTTCCTCCTTTTGTAAC	NheI	
Stab185	F	GCAGCGGCCGCATGAATGAGTGGTATGCT	NotI	177
	R	GGTGCTAGCTTATCTCTCCTTATCAAATTCTT	NheI	
Stab187	F	GCAGCGGCCGCATGAAGCAGAGAGATTTTG	NotI	291
	R	GGTGCTAGCTTAAATATCTAATTTCTCATAACAAT	NheI	
Stab188	F	GCAGCGGCCGCATGAACAAAGCAGTAGAA	NotI	285
	R	GGTGCTAGCCTACTTTATAAAACCTTTAAGTTC	NheI	
Stab190	F	GCAGCGGCCGCATGAATGGTATTATTGTATTTTAC	NotI	303
	R	GGTGCTAGCTTATTGACTCATCTCCTCTAA	NheI	
Stab191	F	GCAGCGGCCGCATGGTAATTGCGTTTTTTAT	NotI	402
	R	GGTGCTAGCTCACTCCTTATTAAGTTCAATT	NheI	
Stab192	F	GCAGCGGCCGCATGGAATTTATAGATAAAAATAATGT	NotI	234
	R	GGTGCTAGCTCATAGTATGTCCTCCTTTTT	NheI	
Stab194	F	GCAGCGGCCGCGTGGAGAAATTCAAAGGT	NotI	318
	R	GGTGCTAGCTTATTTCCCTCCTTCAATCT	NheI	
Stab195	F	GCAGCGGCCGCATGGAATATTTATTTTTATTTATAGG	NotI	228
	R	GGTGCTAGCTTAAAAGAATAAAATCTTAATTTCTT	NheI	
Stab196	F	GCAGCGGCCGCATGAAACATTTTATTTTAATTTTAGG	NotI	177
	R	GGTGCTAGCTTAATTTCTACTAAACATACTTCC	NheI	
Stab198	F	GCAGCGGCCGCATGAAAGTAGAATCAATAGCA	NotI	315
	R	GGTGCTAGCTTATTTTTCCTCCTTAAAATATCTT	NheI	
Stab199	F	GCAGCGGCCGCGTGTCTAATAAAACTATTACAAA	NotI	678
	R	GGTGCTAGCTTAATTTTTAATGATACCTACTAAT	NheI	
Stab201	F	GCAGCGGCCGCATGAATTATGAAGAGGTACT	NotI	222
	R	GGTGCTAGCTTAAAATAAAATAGCTCCTGC	NheI	
Stab202	F	GCAGCGGCCGCATGAATTATAGAGATTTTATTACAGA	NotI	198
	R	GGTGCTAGCTTATAACCCCTCCGTTGT	NheI	
Stab204	F	GCAGCGGCCGCATGTATCCTGAAATAGATGT	NotI	306
	R	GGTGCTAGCTCATTTTGTTGATAGCTCC	NheI	
Stab206	F	GCAGCGGCCGCATGGTAAAATTAGATAAATACTTAA	NotI	393
	R	GGTGCTAGCTTAGTATTCTCCTTCTGTTATT	NheI	
Stab208	F	GCAGCGGCCGCATGATTTATAAAATATCAAAACATAA	NotI	243
	R	GGTGCTAGCCTATGGCTGTAACCATTC	NheI	
Stab209	F	GCAGCGGCCGCATGATTATAGATAAATTAAATGGAG	NotI	390
	R	GGTGCTAGCCTATTTCTCTCCTTTTAATTCTTT	NheI	
Stab210	F	GCAGCGGCCGCATGAGTAATAGTTGGGAAAAA	NotI	195
	R	GGTGCTAGCTTATTTATCTGCTACATACTCAT	NheI	
Stab211	F	GCAGCGGCCGCATGATGAATGGAAAACAAAT	NotI	294
	R	GGTGCTAGCTTACATACCTTTCACATAGTC	NheI	
Stab212	F	GCAGCGGCCGCATGAAAAAACTATTAATATTATTTAC	NotI	309

	R	GGTGCTAGCTTAATCTCCTTTATATATTAATTCAT	NheI	
Stab213	F	GCAGCGGCCGCATGTATATATTAGAAAGAACAATTAG	NotI	237
	R	GGTGCTAGCTCATAAGTCATTCTCCCAC	NheI	
Stab215	F	GCAGCGGCCGCATGATAAATATAGAACATGATTATAC	NotI	192
	R	GGTGCTAGCTTACCATCGTTCAATAGATAC	NheI	
Stab216	F	GCAGCGGCCGCATGAATGCTAGGAAAGCA	NotI	351
	R	GGTGCTAGCTTACCAACTAATGTATATAATAGGT	NheI	
Primer pairs for cloning 16 potential toxic HPUFs into pBAD33.				
Stab008	F	AGCCTGGGTACCAGGAGGAAACGATGGTTACTTTAACATACACTAT T	KpnI	204
	R	AGCCTGTCTAGACTATCCTACGTGCCAAGC	XbaI	
Stab024	F	AGCCTGGGTACCAGGAGGAAACGATGAACATTAACGAATATATAGG	KpnI	657
	R	AGCCTGTCTAGACTACCTCCCTAAGTCTTTT	XbaI	
Stab062c	F	AGCCTGGGTACCAGGAGGAAACGATGGTAAATAAAATTAACGATAA A	KpnI	624
	R	AGCCTGTCTAGATTATCCATCTTGTTCCCC	XbaI	
Stab075c	F	AGCCTGGGTACCAGGAGGAAACGATGGCACTACTTTTAACAT	KpnI	309
	R	AGCCTGTCTAGATTACATTTCTCCTTTTTCTATTT	XbaI	
Stab081c	F	AGCCTGGGTACCAGGAGGAAACGTTGGATAAGGAGATAAACAAC	KpnI	333
	R	AGCCTGTCTAGACTATGCAAATTTGTTAAAGACA	XbaI	
Stab085	F	AGCCTGGGTACCAGGAGGAAACGATGAAAACAAAGAAAGAAATTAA A	KpnI	279
	R	AGCCTGTCTAGATCAATCCATTTCACCTCG	XbaI	
Stab156	F	AGCCTGGGTACCAGGAGGAAACGATGGGAATTATAGTAAACTCC	KpnI	756
	R	AGCCTGTCTAGATTACTCATAACTGCTTCCTT	XbaI	
Stab159	F	AGCCTGGGTACCAGGAGGAAACGATGAAGTTCAATGATATTTATGA	KpnI	312
	R	AGCCTGTCTAGACTATAAGAAATCCTTTTCCATTTTT	XbaI	
Stab172	F	AGCCTGGGTACCAGGAGGAAACGATGGTGAGTAAATTTATCGG	KpnI	252
	R	AGCCTGTCTAGATTATTCATTTTCTTTATCCTTAATG	XbaI	
Stab175	F	AGCCTGGGTACCAGGAGGAAACGATGATAAGCTCATTTGATAGT	KpnI	249
	R	AGCCTGTCTAGACTATAGTAAAATATTGTTTACTGCT	XbaI	
Stab187	F	AGCCTGGGTACCAGGAGGAAACGATGAAGCAGAGAGATTTTG	KpnI	294
	R	AGCCTGTCTAGATTAAATATCTAATTTCTCATAACAAT	XbaI	
Stab209	F	AGCCTGGGTACCAGGAGGAAACGATGATTATAGATAAATTAAATGG AG	KpnI	393
	R	AGCCTGTCTAGACTATTTCTCTCCTTTTAATTCTTT	XbaI	
Stab212	F	AGCCTGGGTACCAGGAGGAAACGATGAAAAAACTATTAATATTATT TAC	KpnI	312
	R	AGCCTGTCTAGATTAATCTCCTTTATATATTAATTCAT	XbaI	
Stab213	F	AGCCTGGGTACCAGGAGGAAACGATGTATATATTAGAAAGAACAAT TAG	KpnI	240
	R	AGCCTGTCTAGATCATAAGTCATTCTCCCAC	XbaI	
Stab215	F	AGCCTGGGTACCAGGAGGAAACGATGATAAATATAGAACATGATTA TAC	KpnI	195
	R	AGCCTGTCTAGATTACCATCGTTCAATAGATAC	XbaI	
Stab216	F	AGCCTGGGTACCAGGAGGAAACGATGAATGCTAGGAAAGCA	KpnI	354
	R	AGCCTGTCTAGATTACCAACTAATGTATATAATAGGT	XbaI	
Primers for the confirmation of gene insertion in pBAD33				
BadFor		CTACCTGACGCTTTTTATCGCAAC		204
BadRev		GCAAATTCTGTTTTATCAGACCGC		
Primers for pRAB11N construction.				

pRAB-fw		TCTCTATCATTGATAGAGTATGATGGTACCGTTAACAGATCTGAGCTC	KpnI, BglII	55
pRAB-rev		GCTTATTTTAATTATACTCTATCAATGAT		
Primers for the confirmation of construction of and gene insertion in pRAB11N				
pRAB11-F		TCTCTATCATTGATAGAGTATGAT		
fR-346		CAGATTGTACTGAGAGTGCACCA		
Primer pairs for cloning 9 potential toxic HPUFs into pRAB11N.				
Stab024	F	AGCCTGGGTACCAGGAGGAAACGATGAACATTAACGAATATATAGG	KpnI	657
	R	AGCCTGAGATCTCTACCTCCCTAAGTCTTTT	BglII	
Stab081c	F	AGCCTGGGTACCAGGAGGAAACGTTGGATAAGGAGATAAACAAC	KpnI	333
	R	AGCCTGAGATCTCTATGCAAATTTGTTAAAGACA	BglII	
Stab159	F	AGCCTGGGTACCAGGAGGAAACGATGAAGTTCAATGATATTTATGA	KpnI	312
	R	AGCCTGAGATCTCTATAAGAAATCCTTTTCCATTTTT	BglII	
Stab172	F	AGCCTGGGTACCAGGAGGAAACGATGGTGAGTAAATTTATCGG	KpnI	252
	R	AGCCTGAGATCTTTATTCATTTTCTTTATCCTTAATG	BglII	
Stab175	F	AGCCTGGGTACCAGGAGGAAACGATGATAAGCTCATTTGATAGT	KpnI	249
	R	AGCCTGAGATCTCTATAGTAAAATATTGTTTACTGCT	BglII	
Stab187	F	AGCCTGGGTACCAGGAGGAAACGATGAAGCAGAGAGATTTTG	KpnI	294
	R	AGCCTGAGATCTTTAAATATCTAATTTCTCATAACAAT	BglII	
Stab209	F	AGCCTGGGTACCAGGAGGAAACGATGATTATAGATAAATTAAATGGAG	KpnI	393
	R	AGCCTGAGATCTCTATTTCTCTCCTTTTAATTCTTT	BglII	
Stab212	F	AGCCTGGGTACCAGGAGGAAACGATGAAAAAACTATTAATATTATTTAC	KpnI	312
	R	AGCCTGAGATCTTTAATCTCCTTTATATATTAATTCAT	BglII	
Stab213	F	AGCCTGGGTACCAGGAGGAAACGATGTATATATTAGAAAGAACAATTAG	KpnI	354
	R	AGCCTGAGATCTTCATAAGTCATTCTCCCAC	BglII	
Primers for toxic control gene ORF104				
f77tox-F		AGCCTGGGTACCAGGAGGAAACGATGGTAACCAAAGAATTTTTAAAAAC	KpnI	158
f77tox-R		AGCCTGAGATCTTTAATATTCGACGATAGCGGG	BglII	
