# Supplementary material for: Discovery of Bactericidal Proteins from Staphylococcus Phage Stab21 Using a High-Throughput Screening Method
Source: Antibiotics (Basel). 2023 Jul 21;12(7):1213. doi: 10.3390/antibiotics12071213 (PMC10376165; doi:10.3390/antibiotics12071213)
Supplement: Supplementary file 1 [file antibiotics-12-01213-s001.zip › Table S4.pdf]

**Table S4.** Gene inducing and non-inducing pair conditions tested for *E. coli* and *S. aureus* toxicity drop test using recombinant strains. Gene expression is induced in the presence of arabinose (Ara) or anhydrotetracycline (ATc). Antibiotics were added during the preparation of tryptic soy agar plates. Cm10, chloramphenicol 10 μg/mL; Cm30, Cm 30 μg/mL; Amp100, ampicillin 100 μg/mL.

| **Strains** | **Non-inducing conditions** | **Inducing conditions** |
|---|---|---|
| *E. coli* | | |
| DH5α/pBAD33-HPUFs | Amp100 + Glucose 0.2 % | Amp100 + Ara 0.2 % |
| DH10B/pRAB11N-HPUFs | Amp100 | Amp100 + ATc 0.4 μM |
| *S. aureus* | | |
| RN4220/ pRAB11N-HPUFs | No antibiotics | ATc 0.1 μM |
| RN4220/ pRAB11N-HPUFs | No antibiotics | ATc 0.2 μM |
| RN4220/ pRAB11N-HPUFs | No antibiotics | ATc 0.3 μM |
| RN4220/ pRAB11N-HPUFs | No antibiotics | ATc 0.4 μM |
| RN4220/ pRAB11N-HPUFs | Cm10 | Cm10 + ATc 0.4 μM |
| RN4220/ pRAB11N-HPUFs | Cm30 | Cm30 + ATc 0.1 μM |
| RN4220/ pRAB11N-HPUFs | Cm30 | Cm30 + ATc 0.2 μM |
| RN4220/ pRAB11N-HPUFs | Cm30 | Cm30 + ATc 0.4 μM |
