# Supplementary figures and images for: Discovery of Bactericidal Proteins from Staphylococcus Phage Stab21 Using a High-Throughput Screening Method
Source: Antibiotics (Basel). 2023 Jul 21;12(7):1213. doi: 10.3390/antibiotics12071213 (PMC10376165; doi:10.3390/antibiotics12071213)

**Figure S1.** Relative joint-sequence reads ratios for non-toxic genes from Stab21 phage.

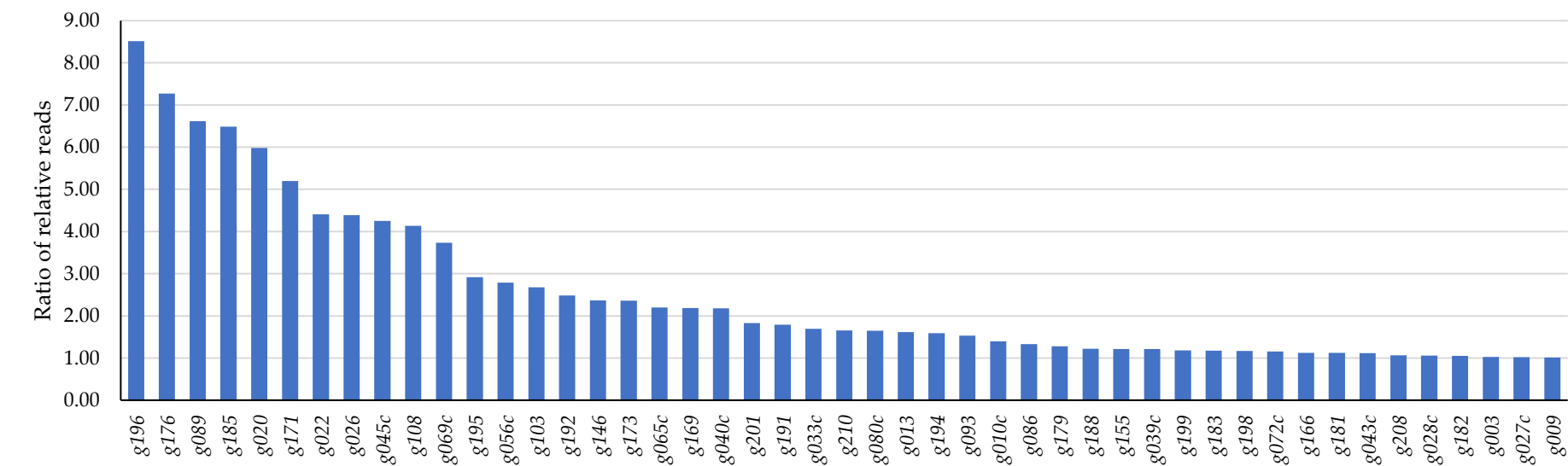

Supplement: Supplementary file 1 [file antibiotics-12-01213-s001.zip › Figure S1.pdf]
